# Prospective mixture risk assessment and management prioritizations for river catchments with diverse land uses

**DOI:** 10.1002/etc.3960

**Published:** 2018-02-12

**Authors:** Leo Posthuma, Colin D. Brown, Dick de Zwart, Jerome Diamond, Scott D. Dyer, Christopher M. Holmes, Stuart Marshall, G. Allen Burton

**Affiliations:** ^1^ National Institute for Public Health and the Environment (RIVM) Centre for Sustainability Environment and Health Bilthoven The Netherlands; ^2^ Department of Environmental Science Institute for Wetland and Water Research Faculty of Science Radboud University Nijmegen The Netherlands; ^3^ Environment Department University of York Heslington York UK; ^4^ Mermayde Groet The Netherlands; ^5^ Tetra Tech Owings Mills Maryland USA; ^6^ The Procter and Gamble Company Cincinnati Ohio USA; ^7^ Waterborne Environmental Leesburg Virginia USA; ^8^ Safety and Environmental Assurance Centre Unilever Sharnbrook Bedford United Kingdom; ^9^ School for Environment and Sustainability University of Michigan Ann Arbor Michigan USA

**Keywords:** Chemical mixture, Aquatic risk assessment, Watershed management, Catchment assessment, Exposure scenario, Ecological risk assessment

## Abstract

Ecological risk assessment increasingly focuses on risks from chemical mixtures and multiple stressors because ecosystems are commonly exposed to a plethora of contaminants and nonchemical stressors. To simplify the task of assessing potential mixture effects, we explored 3 land use–related chemical emission scenarios. We applied a tiered methodology to judge the implications of the emissions of chemicals from agricultural practices, domestic discharges, and urban runoff in a quantitative model. The results showed land use–dependent mixture exposures, clearly discriminating downstream effects of land uses, with unique chemical “signatures” regarding composition, concentration, and temporal patterns. Associated risks were characterized in relation to the land‐use scenarios. Comparisons to measured environmental concentrations and predicted impacts showed relatively good similarity. The results suggest that the land uses imply exceedances of regulatory protective environmental quality standards, varying over time in relation to rain events and associated flow and dilution variation. Higher‐tier analyses using ecotoxicological effect criteria confirmed that species assemblages may be affected by exposures exceeding no‐effect levels and that mixture exposure could be associated with predicted species loss under certain situations. The model outcomes can inform various types of prioritization to support risk management, including a ranking across land uses as a whole, a ranking on characteristics of exposure times and frequencies, and various rankings of the relative role of individual chemicals. Though all results are based on in silico assessments, the prospective land use–based approach applied in the present study yields useful insights for simplifying and assessing potential ecological risks of chemical mixtures and can therefore be useful for catchment‐management decisions. *Environ Toxicol Chem* 2018;37:715–728. © 2017 The Authors. Environmental Toxicology Chemistry Published by Wiley Periodicals, Inc.

## INTRODUCTION

The present study is an output of a Society of Environmental Toxicology and Chemistry (SETAC) Pellston workshop®, “Simplifying Environmental Mixtures —An Aquatic Exposure–Based Approach Via Exposure Scenarios,” which was held in March 2015 with the aim of looking at 1) whether a simplified scenario‐based approach could be used to help determine if mixtures of chemicals posed a risk greater than that identified using single chemical–based approaches and 2), if so, what might be the magnitude and temporal aspects of the exceedances so as 3) to determine whether the application of the approach provides insights in mixtures of greatest concern and the compounds dominating those mixtures (prioritization). The aims of the present study were to combine the land‐use scenarios of the associated manuscripts of the Pellston workshop, references Holmes et al. (2018), Diamond et al. (2018), and de Zwart et al. (2018), to investigate these questions for catchments with different combinations of land use.

The goal of various environmental policies in human‐dominated ecosystems is to achieve a nontoxic environment and sound biological integrity (European Commission). This status has not been reached in many freshwater and marine systems, based on evidence on the occurrence of a wide array of chemicals in surface waters (Bradley et al. 2017) and organisms' tissues (US Environmental Protection Agency 2009), with associated evidence for multiple contaminant risks (Malaj et al. 2014), impacts in bioassays (Conley et al. 2017), and reduced species biodiversity and abundance in various human‐dominated systems (Schäfer et al. 2016; Posthuma et al. 2016). Achieving negligible exposures and nontoxic conditions is challenging given the multitude of chemicals associated with human sources such as agricultural practices, treated wastewater, and urban runoff. Currently produced chemicals may cause direct species loss but also effects such as fish intersex and possibly other unknown effects (Kolpin et al. 2002), and new chemicals are continuously produced and emitted (Gessner and Tlili 2016). Regulatory approaches regarding chemicals presently focus, however, on a relatively small number of chemicals for which there are established environmental quality standards (EQS). Less is known about how to assess and reduce the risks and effects of ambient mixtures.

The assessment and management of ecological risk for a highly complex matrix of combinations of chemicals, sites, species, and ecosystems can proceed via various approaches. The traditional approach is based on risk assessment of individual chemicals, using generic protective EQS. Those are benchmark concentrations, such as the predicted no‐effect concentration (PNEC). A predicted or measured environmental concentration (PEC or MEC) below such a threshold is interpreted as protective of ecosystem structure and function, that is, the risk quotient (RQ = PEC/benchmark concentration or RQ = MEC/benchmark concentration) is <1. The origin of these methods dates back to the 1970s and 1980s (Stephan et al. 1985; Van Straalen and Denneman 1989). Since then tailored methods have been defined to serve specific policy goals, such as generic water quality policies and policies to determine the environmental hazards of plant protection products (PPPs) for aquatic edge‐of‐field exposures (Geiser 2015). Recently, chemical mixture assessment approaches have been recommended for practical application (Kortenkamp et al. 2009). Many of these mixture approaches evaluate mixture risks by a default approach via aggregation of the individual RQs for each chemical in the mixture, such as the hazard index (HI = ∑RQ = ∑[PEC/benchmark concentration]), although the expected mixture effects are also quantified via mixture toxic pressures for species assemblages, expressed as multisubstance potentially affected fraction (msPAF) of species (de Zwart and Posthuma 2005). In addition, various methods are available to retrospectively evaluate the ecological risks and impacts of mixtures on the landscape scale (Posthuma et al. 2016). The latter approaches offer an a posteriori quantitative risk or impact ranking of sites and stressors of concern (including chemical mixtures).

In the present study we describe a prospective analysis of land use–related emissions, exposures, and risks of chemical mixtures. This concerns both the resulting chemical signatures (are there land use–specific mixture compositions [Holmes et al. 2018; Diamond et al. 2018; de Zwart et al. 2018]?) as well as the resulting chemical footprints (is there a net risk exported from a catchment to a downstream water body [Zijp et al. 2014; Bjørn et al. 2014]?). Prospective, catchment‐scale prioritization of chemical mixture risks can assist decision‐making regarding risk‐mitigation strategies (Ginebreda et al. 2013; Coppens et al. 2015; Sobek et al. 2016; Brack et al. 2017). The present study expands on and integrates 3 detailed analyses of land use–related scenarios, investigating the specific chemical signatures of an agriculture scenario (emissions from agricultural land dictated by rainfall, soils, and PPP use [Holmes et al. 2018]), a treated domestic wastewater scenario (daily use of household chemicals [Diamond et al. 2018]), and an urban runoff scenario (rainfall‐mediated emissions from city surface areas [de Zwart et al. 2018]).

The goal of the present study was to develop and test the utility of combining the concepts of continuous exposure of treated domestic wastewater discharge with temporally variable chemical exposure scenarios associated with urban and agricultural land uses for the purpose of supporting comprehensive mixture risk assessments and environmental management. To achieve this, the following objectives were addressed: 1) propose and evaluate an approach for deriving a likely chemical signature in a receiving river catchment to help explain field observations (concentrations and/or impacts) and provide a background against which the toxicity of a new product or a new usage could be assessed, 2) produce an approach balancing pragmatism and simplicity with adequate detail for a scientifically credible outcome, 3) recognize the complexity of assessing both the exposure and effects of mixtures and derive generalizations that provide evidence for a reality check of ecological risk assessment, and 4) identify uncertainties and gaps in knowledge requiring further research to refine the prospective assessment of chemical mixtures.

## COMBINED SCENARIOS

### Overall approach

We integrated risk‐assessment approaches for 3 typical human‐based emission scenarios (agriculture, domestic, urban runoff) and focused on identifying the potential for mixture effects in receiving waters. The scenarios were selected because they commonly occur in human‐dominated systems and differ vastly in their chemical emission characteristics. The scenarios were further developed and substantiated as land‐use scenarios, whereby domestic and urban runoff are combined as the land use CITY. Further, the land use nature was added for demonstrating the influence of water inputs within the catchment where chemical emissions are negligible. The scenarios were combined in a catchment‐assessment model, with the option to define land uses for between 1 and 10 subcatchments. Their integration placed the different single land‐use categories into a landscape‐level perspective. This allowed for cross‐comparisons and integrated exposure and risk analyses, to evaluate the utility and limitations of land‐use scenarios for environmental assessment and potential management of chemical mixtures.

### Modeling land uses, geography, and hydrology

**Figure 1:**
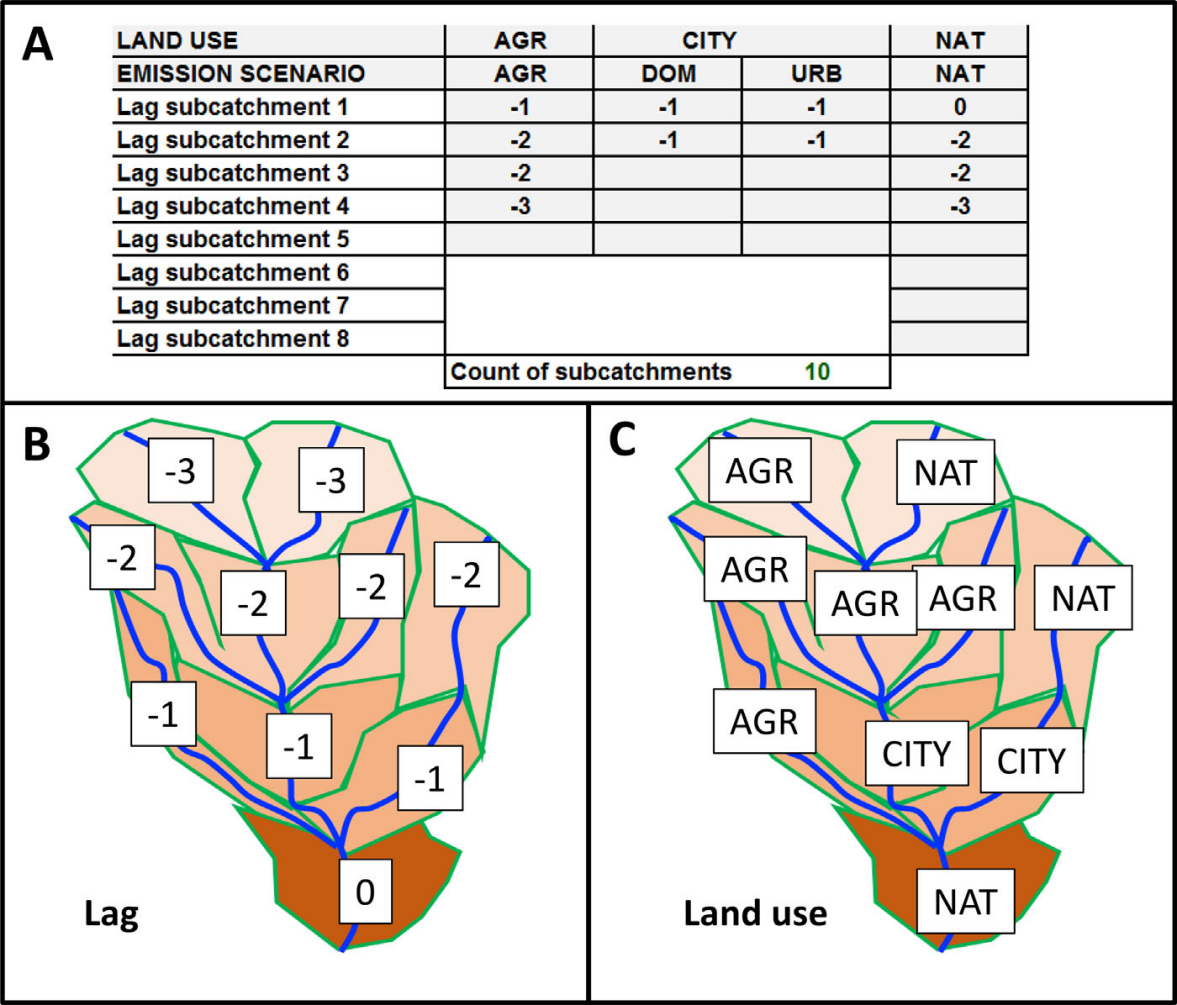
The MIXED land‐use scenario layout of 10 subcatchments of similar size (10 km^2^ each) in a total catchment of maximally 100 km^2^. Water flows from the top of the figure to the bottom. (**A**): Scenario definition table, defining the catchment, with land use and associated emission types. Bottom: Resulting catchment map with position codes (related to lag times of flow, (**B**)) and land‐use codes (**C**) as defined in the scenario definition table. The different color intensities of the subcatchments indicate various hydrological travel times to reach the main catchment outlet, which enables modeling of time‐dependent chemical fate processes. Other scenarios can be defined via entering codes for the lag times of the land uses in the scenario definition table. AGR = agriculture; DOM = domestic; NAT = nature; URB = urban runoff.

The scenarios agriculture, CITY (domestic +** **urban runoff), and nature were spatially combined in hypothetical but realistic spatial arrangements to represent either a single–land use scenario in a subcatchment or a catchment with multiple land uses and river confluences. A spreadsheet model represented the various catchment layouts. The model included hydrology, aquatic emissions, concentrations, and mixture assessment outcomes for (in its most complex format) a catchment of 100 km^2^ with 10 subcatchments of 10 km^2^ each, linked within a river network (Figure 1). A subcatchment was defined to have only one land use. A catchment can have any combination and number of subcatchments (in our case, up to 10) and assigned land uses. The land uses shown in Figure 1 define the layout of the modeled MIXED land‐use scenario, which is just one of many possible catchment layouts.

The characteristics of the separate emission scenarios (agriculture, domestic, urban runoff, and nature) were developed based on literature reviews and by combining hydrological and ecotoxicological modeling techniques with regulatory judgment criteria (Table 1). Each scenario layout was modeled for 20 yr, with daily quantifications of PECs for the each of the studied chemicals. Details are in the Supplemental Data (section 1) and the scenario reviews (Holmes et al. 2018; Diamond et al. 2018; de Zwart et al. 2018).

**Table 1:** Characteristics of the original land‐use scenario studies (Holmes et al. 2018, Diamond et al. 2018, de Zwart et al. 2018) and evaluations based on a subcatchment area of 10 km^2^

	Agriculture	Domestic	Urban runoff	Nature
Emissions	Rain event and PPP use–related (discontinuous, PPP use related to crop type)	Household‐related (continuous, household chemicals, WWTP‐chemical removal efficacies in Supplemental Data, Table S1)	Rain event–related (discontinuous, from wearing of buildings, brake pads, oils, etc.)	None
Emissions source	13 PPPs applied annually to winter wheat	Typical no. people/area, (10 000 inhabitants) Water use 200 L/person/d Effluent flow 0.0231 m^3^/s	Runoff, occurring at >10.3 mm rainfall/d (P95 of rainfall)	None
Chemicals	Boscalid Chlorothalonil Cypermethrin Epoxiconazole Flufenacet FluoxastrobinIodosulfuron‐methyl Mesosulfuron‐methyl Pendimethalin Prochloraz Proquinazid Prothioconazole Pyraclostrobin	1‐OH‐Benzotriazole Acesulfame Benzalkonium chloride Caffeine Carbamazepin Erythromycin Sulfomethoxazole Ethinylestradiol HHCB (galaxolide) Ibuprofen Linear alkylbenzene sulfonate Methylisothiazolinone TiO Zinc acetate Zinc oxide	Aluminium Benz[*a*]anthracene Bifenthrin Copper (dissolved) Deltamethrin Fluoranthene Iron (dissolved) Nonylfenolmonoethoxylate Permethrin Zinc (dissolved)	
Benchmark for PECs	Tier 1: RAC Tier 2: RAC species groups	Tier 1: PNEC Tier 2: PNEC of species groups	Tier 1: median EC50 (all species)	
Assessing mixtures	∑PEC/RAC^a^	∑RCR^a^	∑RC^a^ msPAF_EC50_	
Reference	Holmes et al. 2018	Diamond et al. 2018	de Zwart et al. 2018	

^a^ In the present study a predicted environmental concentration benchmark ratio is generally referred to as the hazard index.

EC50 = median effect concentration; HHCB = 1,3,4,6,7,8‐hexahydro‐4,6,6,7,8,8,‐hexamethyl‐cyclopenta[g]benzopyran; PEC = predicted environmental concentration; PNEC = predicted‐no‐effect concentration, utilized in generic protective chemical regulations; PPP = plant protection product; RAC = regulatory acceptable concentrations for edge‐of‐field water bodies, utilized in PPP regulations; RCR = risk characterization ratio (similar to hazard index in the present study); msPAF_EC50_ = multisubstance potentially affected fraction of species exposed beyond their EC50; WWTP = wastewater treatment plant.

### Modeling concentrations

Emissions of chemicals from agriculture, domestic, and urban runoff were derived from individual land‐use studies (details in those reviews and the Supplemental Data). The agriculture scenario incorporated time dependency of emissions related to PPP use on row crops. A 20‐yr time period was modeled on a daily basis by using actual pesticide usage application data for a large arable farm in eastern England (see Holmes et al. 2018) and actual rain events from the FOCUS R1 scenario meteorological data set (used in European Union regulatory modeling for PPPs), which is directly applicable to United Kingdom agricultural conditions. The selected agriculture scenario used a winter wheat exposure scenario, with 13 active ingredients applied on known dates and rates. Accordingly, the scenarios for the other emissions (domestic, urban runoff) were reformulated to enable modeling for the same 20‐yr period and combined into the spreadsheet model. Emission data and hydrological data were combined to estimate concentrations for each of the studied chemicals emitted from each of the land uses.

The spreadsheet model allowed the prediction of concentrations from agriculture, domestic, and urban runoff emissions separately as well as their combinations based on the subcatchment configuration (Figure 1). The model yields 24‐h PECs for subcatchment outlets. Large numbers of PECs were calculated using this approach. For example, for agriculture the number of PECs equals 94 198 (7246 d, 13 chemicals) and for MIXED, 268 102 (7246 d, 37 compounds).

### Risk‐assessment methodologies and prioritizations

The risk patterns associated with the PECs were explored using 3 approaches: HIs, maximum cumulative ratios (MCRs; Vallotton and Price 2016), and mixture toxic pressures (multisubstance potentially affected fraction of species, [msPAF; de Zwart and Posthuma 2005]). Details are in Supplemental Data (section 2).

First, the risks posed by a mixture were determined using individual chemical hazard quotients (HQs) and the net HI, in which HQ_*ij*_ = PEC_*i*_/BM_*ij*_ (with *i* = substance, *j =* selected effect endpoint, with *j* defined as regulatory EQS, chronic no‐observed‐effect concentration [NOEC], or acute median effect concentration [EC50], or regulatory acceptable concentration (RAC) see below), and HI_*j*_ = ΣHQ_*ij*_. The HI is the sum of the individual values of compound‐related HQs, implying the use of concentration additivity as a default mixture model.

Second, the MCR is the maximum cumulative ratio posed by a combined exposure to multiple chemicals under the assumption of concentration addition divided by the risk of the most toxic compound of the sample. The MCR of a sample expresses whether the net predicted toxicity is driven by multiple components which make a significant contribution to the net mixture toxicity. The MCR‐value of a sample was calculated as the ratio of the sample's HI and the highest value of the sample's set of values: MCR = HI/max(HQ). The combination of HI and MCR was used to create subgroupings of the 7246 time samples per scenario in 4 groups: groups I, II, IIIA, and IIIB (Table 2).

**Table 2:** Definition of sample subgroups at the outlet of the (sub‐)catchment, characterized by grouping the maximum cumulative ratios (Vallotton and Price 2016)

Group	Mixture risk (HI)	Individual risk (HQ)	MCR	Meaning
I	HI >1	Max HQ >1		Mixture presents potential risk already based on individual compounds
II	HI <1	Max HQ <1		Assessment does not identify a concern
IIIA	HI >1	Max HQ <1	MCR <2	Mixture risk arises only from summing individual substance risk, although the majority of the mixture risk is driven by one substance
IIIB	HI >1	Max HQ <1	MCR >2	Mixture risk arises only from summing individual substance risk, with overall risk driven by multiple components

HI = hazard index; HQ = hazard quotient; MCR = maximum cumulative ratio.

The HI‐MCR method was applied using different benchmark definitions to derive the HI, representing different tiers and meanings. For tier 1, HIs were defined by generic, protective regulatory criteria (the annual average EQS [AA‐EQS] of the European Water Framework Directive). For tier 2, HIs were defined via the 5th percentile of the species sensitivity distribution (SSD) of chronic NOECs and the 50th percentile of the SSD of EC50s. For tier 3, the MCR was plotted against the mixture toxic pressure (msPAF), derived from the SSD models (SSD_NOEC_ and SSD_EC50_, respectively). In tier 1, HI >1 indicates regulatory concern, whereby it remains uncertain whether direct ecotoxicological effects are likely, for example, because of underlying application factors. In tiers 2 and 3, HI >1 is interpreted as a signal for direct chronic or acute effects on species assemblages, while these HIs have no maximum. In tier 3, in addition, the predicted mixture impact is maximized to 100% of species affected at a chronic or an acute level, respectively. The MCR axis is interpreted as to the number of compounds contributing to the mixture risk.

The scenario results were also summarized as chemical footprints (Zijp et al. 2014). A chemical footprint expresses whether the net emissions in a landscape remain within a preset boundary on risks or effects, for example, the mixture exposure level at which 95% of the species is protected against exceedance of their no‐effect level for the mixture (msPAF_NOEC_ <0.05). In the present study, the approach is modified to summarize the percentage of days the latter is exceeded at the outflow of a subcatchment based on the P95 of the msPAF_NOEC_ of all days of a scenario run.

## RESULTS

### Rainfall and flow

The natural rainfall varied over time and resulted in variation in flow. The vast numbers of input data on rain and output data generated on flow (7246 per scenario) are summarized in Supplemental Data (section 3). The outputs show that the variation in flow implied a strong influence on the dilution of emitted chemical loads and domestic discharge effluents. Summarized as the P99.9/P5 flow ratios, the high to low flow ratios were 55, 324, 128, and 94 for the scenarios CITY, agriculture, nature, and MIXED, respectively.

### PECs

**Figure 2:**
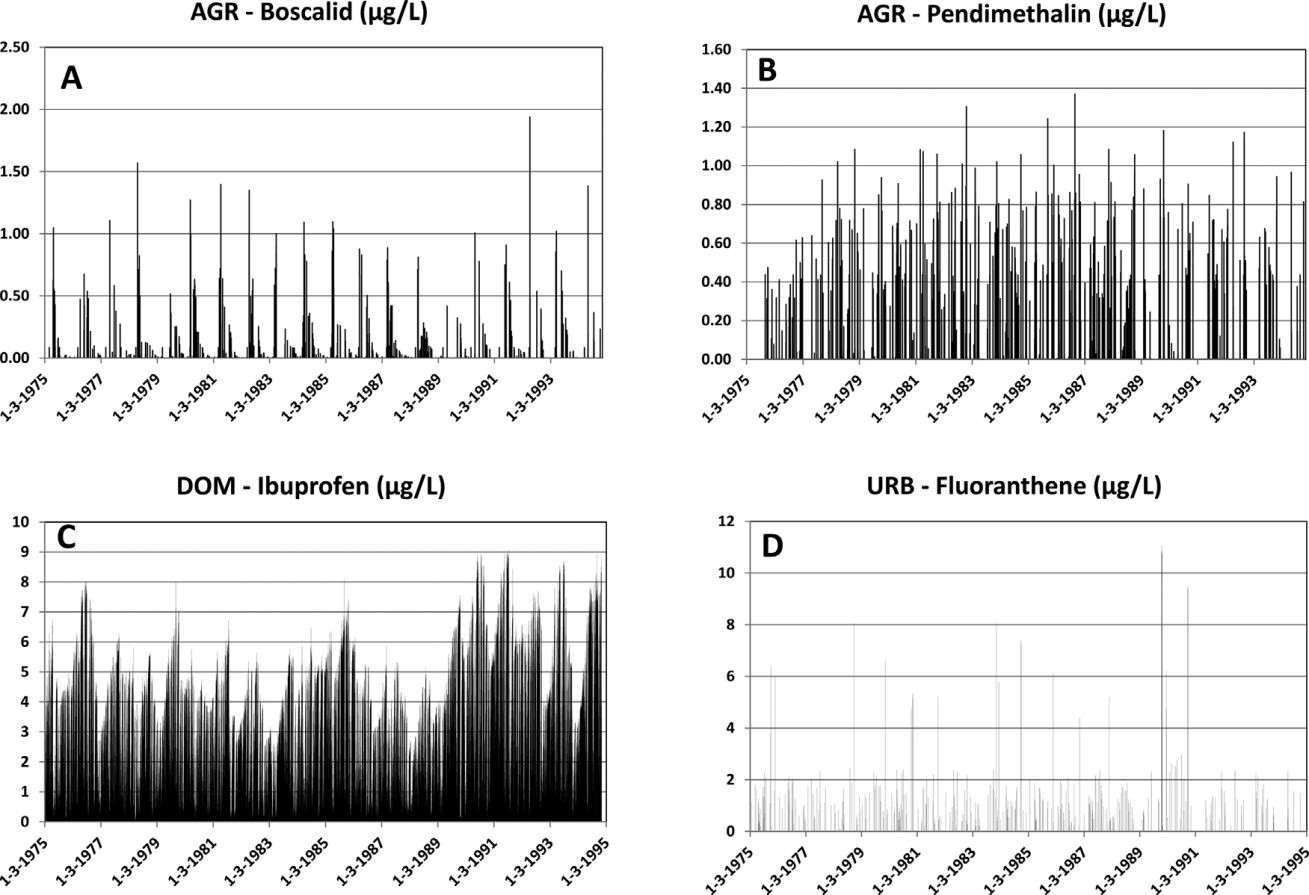
Illustration of the temporal variability of predicted environmental concentrations for 2 substances of the agriculture scenario: boscalid (**A**) and pendimethalin (**B**), one for the domestic scenario: ibuprofen (**C**), and one for the urban runoff scenario: fluoranthene (**D**). AGR = agriculture; DOM = domestic; URB = urban runoff.

The temporal variability of PECs is illustrated in Figure 2. The chemical concentrations varied over time because of the sequential use of PPPs combined with rain events (agriculture) and rain events passing the runoff threshold of 10.3 mm rain (urban runoff). For domestic, though the per capita use of chemicals in this scenario was constant over time, the resulting PECs show spatiotemporal variation because of the effects of variations in hydrological conditions.

### PECs and MECs

**Figure 3:**
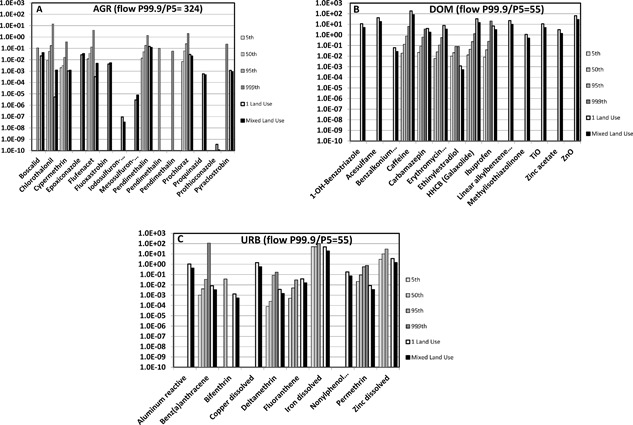
Comparison of measured environmental concentrations (MECs) of surface water systems, summarized as P5, P50, P95, and P99.9, of samples with a detectable concentration (greater than the limit of quantification in the monitoring data) and predicted environmental concentrations (PECs), summarized as P95 at the outflow of a subcatchment. Gray bars, MECs (darkening gray tones from low to high percentiles of detectable MECs); white and black bars, PECs of subcatchments with a single land use and the MIXED scenario, respectively. The P99.9 percentiles are added to demonstrate the magnitude of peak concentrations within the series of 7246 daily PECs per scenario. The flow P99.9/P5 ratio is added to illustrate the magnitude of dilution (PEC) variation related to flow. (**A**) AGR = agriculture; (**B**) DOM = domestic; (**C**) URB = urban runoff.

The PECs were compared to measured values (MECs obtained from available databases and literature (Figure 3; details in Supplemental Data, section 4). Averaged over the chemicals and as represented in the monitoring databases, the fractions of river water samples with measured concentrations higher than the limit of quantification (LOQ) were 1.4% for agriculture, 59.8% for domestic, and 14.1% for urban runoff chemicals. For many field samples (frequency for agriculture > urban runoff > domestic) the MECs were lower than the LOQ. The percentiles of the MEC distributions (Figure 3) therefore refer to the subset of samples with quantifiable concentrations and those of the PECs to the total set of 7246 predicted values for a compound.

For some chemicals, for example, pendimethalin in the agriculture scenario, the upper percentiles of European river water MEC distributions were very similar to the scenario‐based PECs. For other chemicals, the highest MEC percentiles were greater (e.g., chlorothanonil) or lower (e.g., caffeine) than the higher PEC percentiles. Given the flow variation, the degree of similarity between detected MEC percentiles and PEC percentiles suggest that the land‐use scenarios resulted in predicted exposures that may occur in European rivers.

### Risk characterization step 1: PECs and exceedance of regulatory endpoints

Tier‐1 results show that the regulatory benchmark concentrations were exceeded for various subcatchment outlet days and for various compounds (HI >1, see Supplemental Data, Tables S7–S9). Looking at peak exposures (represented by P95‐PEC), the peak PECs of, for example, pendimethalin exceeded the AA‐EQS and the maximum annual concentration EQS (MAC‐EQS) of this compound 8 and 6 times, respectively. For the domestic scenario, the peak exposure of ethinylestradiol and galaxolide exceeded the AA‐EQS 4 and 7.5 times, respectively. For the urban runoff scenario, the highest exceedance was found for deltamethrin, where the peak exposure was 1171 times the standard. Whether exceedances imply ecotoxic effects depends not only on the magnitude but also on the duration of exposure. This also varied. For example, for 7.3, 80, 91, and 5% of the days there was an exceedance of the AA‐EQS of pendimethalin (agriculture), ethinyl estradiol (domestic), galaxolide (domestic), and deltamethrin (urban runoff), respectively. Exposures can thus be shorter or longer and frequent or incidental. These results suggest, from a regulatory perspective, that the river system at the outlet of a subcatchment or the whole catchment was not sufficiently protected, although high values may also result from high HQ values resulting from a high affected fraction related to high uncertainty on the benchment (defining a low benchmark because of high data uncertainty).

### Risk characterization step 2: Characterization of HIs of mixtures

**Figure 4:**
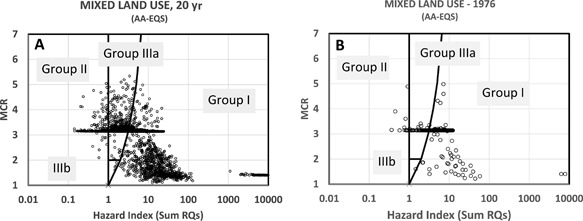
Judgment of the 7246 hazard index (HI)‐maximum cumulative ratio data points for predicted mixtures at the outlet of the whole 100‐km^2^ catchment, according to the land‐use scenario depicted in Figure 1, evaluated by a generically protective regulatory criterion, the annual average environmental quality standard, to define the HI—for all dates (**A**) and for a single (randomly selected) year (**B**). Note: On the left some extremely high HI data points are not shown (see text). AA‐EQS = annual average environmental quality standard; MCR = maximum cumulative ratio; RQ = risk quotient.

The results of tier 1 were summarized as HI‐MCR plots. The MIXED land use (Figure 1) resulted in the plotting of 7246 HI‐MCR data points, which partly overlay each other (Figure 4). The figure suggest that the water at the outflow of the catchment frequently showed HI values (often >>1), which means that the RQs of individual compounds were (far) exceeded, while some of the HI points (with HI >10 000) are not shown. The latter values were found to be related to chemicals of mainly the urban runoff scenario, for days after peak rainfall (causing a runoff event) and for chemicals with low AA‐EQS. The water system is judged to be insufficiently protected for 96% of the days, whereby the MCR remained below 6, with a high frequency of MCR ≅ 3 and many MCRs <3. The theoretical maximum MCR of the MIXED scenario is 37 (when the 37 compounds considered in this scenario are present at equitoxic concentrations, which is unlikely in nature). The relatively low MCRs suggest that a low number of compounds (always fewer than 7) induces HI_AA‐EQS_ >1. The high frequency of similar MCRs at a single level is attributable to a similar change of HI and the maximum HQ of a sample with dilution, because of which HI (X) can vary at nearly constant MCR (Y), whereas the typical HI‐MCR pattern in the CITY scenario related to threshold effects (runoff >10.3 mm rainfall). This threshold contributed to “forcing” the specific pattern of CITY‐MCRs to 2 key MCR levels, related to runoff chemicals' effect criteria.

**Figure 5:**
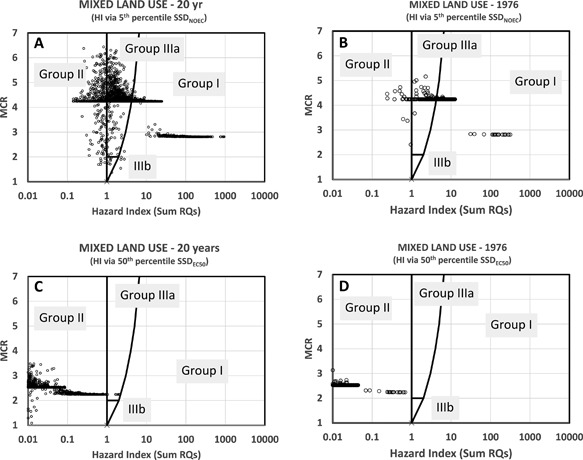
Judgment of the 7246 mixtures at the outlet of the whole 100‐km^2^ catchment, according to the land‐use scenario depicted in Figure 1, judged by compound‐specific hazard quotients derived from the 5th percentile of species sensitivity distribution (SSD) no‐observed‐effect concentrations (**A** and **B**) and the 50th percentile of SSD median effective concentrations (**C** and **D**)—for all dates (**A** and **C**) and for a single (randomly selected) year (**B** and **D**). EC50 = median effective concentration; HI = hazard index; MCR = maximum cumulative ratio; NOEC = no‐observed‐effect concentration; RQ = risk quotient; SSD = species sensitivity distribution.

The tier‐2 analyses resulted in modified HI‐MCR patterns, slightly shifted left for the criterion based on the 95th percentile protection level (Figure 5, upper graphs). Note that both the HI and the MCA of a data point change when the standards underlying the HI change from AA‐EQS to another effect criterion. A tier‐2 evaluation based on EC50s resulted in a further shift of the data points to the left so that only few samples were found where PECs exceeded the EC50 of one or more compounds. Species loss was predicted for those samples, given an earlier observation that msPAF_EC50_ relates to observed species loss in mixture‐exposed aquatic systems (Posthuma and de Zwart 2012). Note that defining another tier‐2 HI using, for example, an EC10 or EC25 as benchmark would result in intermediate shifts (Figure 5, between top and bottom), that is, between chronic exceedance of NOECs and the earliest onset of effects and species loss.

Exposure frequency and time are important in the process of causing ecotoxic effects. Whereas the data points of Figure 5, bottom, may indicate that peak exposures may induce species loss, the same is not true for the data points of Figure 5, top, because those points predict impacts under the condition that chronic exposure occurs. Investigations showed that the exposure times varied across the land uses. For the acute MIXED scenario, the percentage of days and the maximum number of consecutive days for which the mixture exposure HI >1 are 0.1% and 4 d, respectively. The period of high exposure at the outflow of the MIXED scenario is commonly short, but there are a few instances of a few days of exceedance of the mixture EC50. For agriculture, the majority of days where HI_NOEC_ >1 were for a single day. Only on 31 d (0.4%) was the exceedance 2 to 3 d, with no periods of 4 or more days with HI_NOEC_ >1. In short, there was no chronic exposure. The exposure duration differed vastly for CITY, where the majority of days showed HI_NOEC_ >1 (88% of days), and 98% of the exposure lasted at least a consecutive 4 d. The main CITY emission effects were reflected in the exposure durations of the MIXED scenario (HI_NOEC_ >1 for 93% of days, and 86% of exposures lasting at least 4 consecutive days).

### Risk characterization step 3: Mixture toxic pressures

**Figure 6:**
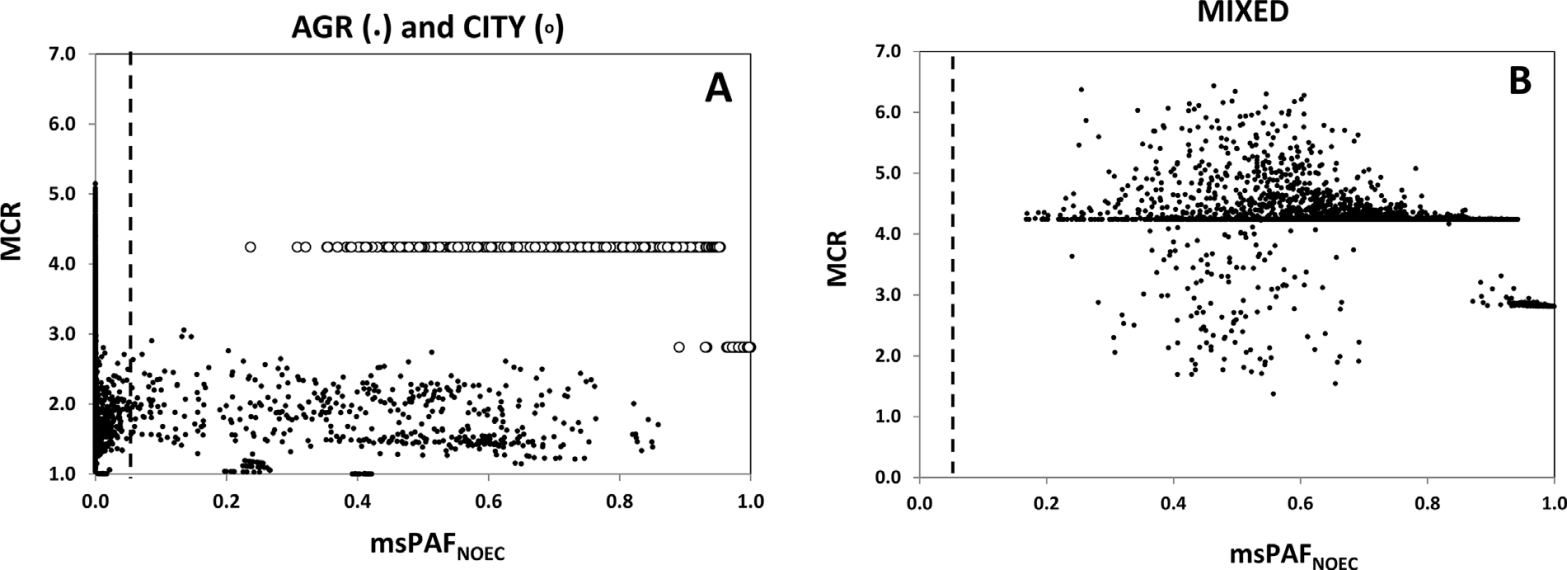
Tier‐3 analyses of mixture impacts in scenarios with (**A**) land use agriculture and CITY (domestic+urban runoff) and (**B**) the MIXED scenario of Figure 1. The dotted line at multisubstance potentially affected fraction no‐observed‐effect concentration (msPAF_NOEC_) = 0.05 is the 95% protection criterion, which was originally used in the derivation of predicted‐no‐effect concentrations for individual compounds. Water samples positioned right of the dotted line are mixture exposures at a level that, if exposure is indeed chronic, induces chronic effects to the fraction of species indicated on the x axis. AGR = agriculture; MCR = maximum cumulative ratio; msPAF = multisubstance potentially affected fraction; NOEC = no‐observed‐effect concentration.

The risk characterization in step 3 consisted of expressing the mixture risks as msPAF_NOEC_ and plotting these outputs again vis à vis the MCAs. The results in Figure 6 suggest that the 95% protection level is exceeded on 8% of the days for agriculture and 100% of the days for CITY (as well as MIXED, not shown), while these chronic toxic pressure levels are associated most often with a few compounds in the mixtures (judged by the MCR values). The CITY and MIXED scenarios consisted of exposures of a chronic kind so that the land use would imply chronic effects for aquatic species assemblages. Acute effects though, quantified via msPAF_EC50_, are more restricted. The maximum acute toxic pressure for agriculture would affect 8% of the species, whereby 1 out of 1000 species would be affected at the peak exposure days (P95 of msPAF_EC50_ ≅ 0.001). For MIXED these values are 63% of the species at the day of the most toxic mixture outflow and 10% of the species at P95.

### Prioritization

Various prioritization analyses can be made to underpin the choice of an abatement scenario aimed at water quality improvement. While in practice “ease of implementation” of abatement measures will be important too, we consider various rationales of risk‐driven prioritization. Details are in Supplemental Data (section 4).

First, prioritization on the basis of land use showed the rank order of mixture risks of CITY (domestic+urban runoff) > agriculture for 3 HI definitions (Table 3). A tier‐1 signal for regulatory concern was most frequent (exposure > AA‐EQS), followed by the frequency of direct sublethal ecotoxic effects (exposure > NOEC), with a low number of modeled samples with species loss of >50%. In the MIXED scenario, prioritizing the maximum HI's using the tier‐2 approach resulted in the mixture risk rank order CITY (urban runoff + domestic) > MIXED >> agriculture (Table 3). The resulting chemical signatures (composition of mixtures and levels of exposure) clearly differ regarding land use.

**Table 3:** Prioritizations on land use, based on various options to define the mixture hazard index

Scenario	Mixture hazard index definition	Signal of	Group I	Group IIIA	Group IIIB	Σ Dates with mixture hazard index >1
AGR	AA‐EQS	Regulatory concern	634	66	14	714
	5th percentile SSD_NOEC_	Sublethal effects	110	46	35	191
CITY (URB+DOM)	AA‐EQS	Regulatory concern	6836	0	355	7191
	5th percentile SSD_NOEC_	Sublethal effects	6577	0	617	7194
MIXED	AA‐EQS	Regulatory concern	4236	4	2710	6950
	5th percentile SSD_NOEC_	Sublethal effects	2442	8	4261	6711
	50th percentile SSD_EC50_	Species loss	0	0	7	7

AA‐EQS = annual average environmental quality standard; AGR = agriculture; DOM = domestic; EC50 = median effect concentration; NOEC = no‐observed‐effect concentration; SSD = species sensitivity distribution; URB = urban runoff.

Second, prioritizations for exposure periods also differ. The agriculture scenario was characterized by peak exposures (always <2 successive days with mixture HI >1), whereas CITY (urban runoff+domestic) and MIXED were characterized by chronically high HIs. Chemical signatures differed regarding exposure dynamics, and even the constant emission of domestic appeared highly dynamic related to hydrology. Further examples are in Supplemental Data, Table S10.

Third, the relative importance of chemicals was assessed. Many prioritizations can be made, for example, for tier 1, 2, or 3 evaluations in each scenario and then on a daily basis (determining the relative importance of each chemical on day = *t*, 7246 times per scenario) or for the numbers of days where the mixture HI >1. Outcomes are in Supplemental Data, Table S10. It appeared that risk prioritization outcomes depend heavily on the tier and inherent risk characterization method. For agriculture, chlorothanonil was, for example, sixth in rank when judged by the AA‐EQS definition of HI but first when judged by chronic SSD_NOEC_‐HI. Again, prioritization needs to account for temporal aspects. Chemicals in domestic would have priority when considering the more chronic character of domestic exposures over urban runoff exposures, while the latter contribute more to the risk of mixtures when present after a runoff event. Comparison to the individual scenario studies demonstrated that the prioritizations shown in Supplemental Data, Table S10, are in line with the outcomes of those scenario studies. For agriculture in the present study, cypermethrin, pendimethalin, and chlorothanonil were found to be important regarding peak exposure levels, ranking first, second, and third, respectively, using AA‐EQS to define HI. Those also ranked high in the agriculture study, with regulatory acceptable concentrations (RAC) as assessment criteria (Holmes et al. 2018). The rankings according to exposure time also showed similar results. The rankings for chronic ecotoxic effects only (present results) identified chlorothalonil and cypermethrin as the first‐ and second‐ranking compounds, which is also in line with the earlier study. For the chemicals emitted in the domestic scenario, the outcomes for galaxolide and ethinylestradiol co‐rank high, although linear alkylbenzene sulfonate ranked lower in the MIXED scenario analyses than in the earlier scenario study (Diamond et al. 2018). For urban runoff, the top‐ranked chemicals were deltamethrin, bifenthrin, permethrin, copper, and zinc, which also rank highly when assessed using landscape scenario analyses (de Zwart et al. 2018). In general, it can be stated that the prioritization options are many, that prioritization outcomes are dynamic in space and time, and, hence, that the problem definition phase should be used to define precisely which ranking information is most valuable for selecting an abatement option. Regulatory prioritization used to prospectively steer preventive policies can thus be different from more realized environmental quality–based rankings (Johnson et al. 2017).

### Chemical footprints

The land‐use scenarios were summarized as chemical footprints for direct, chronic risks for species assemblages. Chemical footprints were quantified using the P95 of the 7246 msPAF_NOEC_ outputs for each scenario (Table 4). A chemical footprint in this definition can be used as management summary information; for example, when the P95‐msPAF_NOEC_ >0.05, this means that for 5% of the days the (sub‐)catchment outflow is ecotoxic such that the 95% protection level is exceeded, whereby a higher degree of exceedance of 0.05 implies a higher potential of the mixtures to affect species assemblages in the downstream water body. In other words, a chemical footprint of 6 for agriculture means that the 95% protection level is exceeded by a factor of 6 or more for 5% of the outflow days. The ecological implication of that depends on exposure time and downstream water body characteristics, although the chemical footprint signals “net outflow of toxicity.” In agriculture, chronic exposures were not found because of the swift effects of the flow regime. In a real system, though, chronic effects related to this chemical footprint may occur when chemicals would slowly accumulate in a water body, for example, in a lentic water body downstream of the outlet.

**Table 4:** Scenarios summarized as chemical footprint indicators

Scenario	P95 msPAF_NOEC_	Chemical footprint (multiplication factor the 95% protection level is exceeded)
AGR	0.30	6.0
AGR–NAT–NAT–NAT	0.14	2.8
CITY	0.95	19.0
CITY–NAT–NAT–NAT	0.93	18.8
MIXED	0.46	9.1
MIXED‐abatement 25%	0.40	8.0
MIXED‐abatement 50%	0.33	6.6
MIXED‐abatement 75%	0.22	4.3

AGR = agriculture; msPAF = multisubstance potentially affected fraction; NAT = nature; NOEC = no‐observed‐effect concentration.

The chemical footprint results ranked the risks of mixtures as CITY > MIXED > agriculture because of higher chemical footprint values and longer exposure durations. An additional scenario—agriculture along a river stretch with three 10‐km^2^ areas with nature downstream (agriculture–nature–nature–nature)—implied a reduction of the chemical footprint compared to agriculture only. For CITY the same layout did not reduce the chemical footprint substantially, related to the fact that the chemical footprint for the CITY scenario (0.95) is at the upper end of an exposure‐mixture risk model which has a sigmoidal shape (like the underlying SSD model) so that a change in chemical emissions induced an equivalent reduction in chemical footprint. As an illustration of the option to evaluate abatement strategies, the bottom lines of Table 4 show changes in chemical footprint following from (imaginary) emission reductions for all chemicals by 25, 50, or 75%. The latter related to only a 47% lowered chemical footprint but an 80% reduction regarding exposure periods for the number of days with HI >1 and 90% for the number of days on which HI >1 was caused by one compound. The 75% abatement option quantified for the MIXED scenario implied that species assemblages at the catchment outflow experience lower exposure peaks, which are much less frequent and more often attributed to a single chemical.

## DISCUSSION

### Overview

The large number of chemicals detected in aquatic environments currently implies that there are large uncertainties regarding whether or not there is sufficient environmental protection against the adverse effects of individual chemicals and their mixtures. The number and diversity of mixtures in the environment seem to imply an intractable number of combinations of exposures, risks, and associated effects, as well as a remaining open end to the problem. This conundrum is often addressed using simplistic approaches (e.g., focusing on priority chemicals) that focus on protection but that ignore mixtures and that use assessment factors to account for the innumerable types of mixtures and uncertainties. However, despite the in silico approach of the present study, the results clearly indicate that the integrated assessment of numerous chemicals with different policy regimes (such as industrial chemicals and PPPs) and spatial–temporal exposure patterns is tractable. Further, the present study demonstrates an application of a strategic tiered approach, which provides refined ecotoxicological insights into the presence of risks for species assemblages (or even specific taxonomic groups, see Holmes et al. 2018). Therefore, the present study presents a testable framework designed to explore simplification and clarification of the spatiotemporal complexity of exposures and provides an approach for forecasting risks based on scenarios created to capture the major influences on exposure for a given catchment or region. The study was based on 3 emission scenario assessments, built into a single approach to model emissions and risks at the scale of realistic combinations of subcatchments and land uses.

### Comparison of predicted and observed parameters

**Figure 7:**
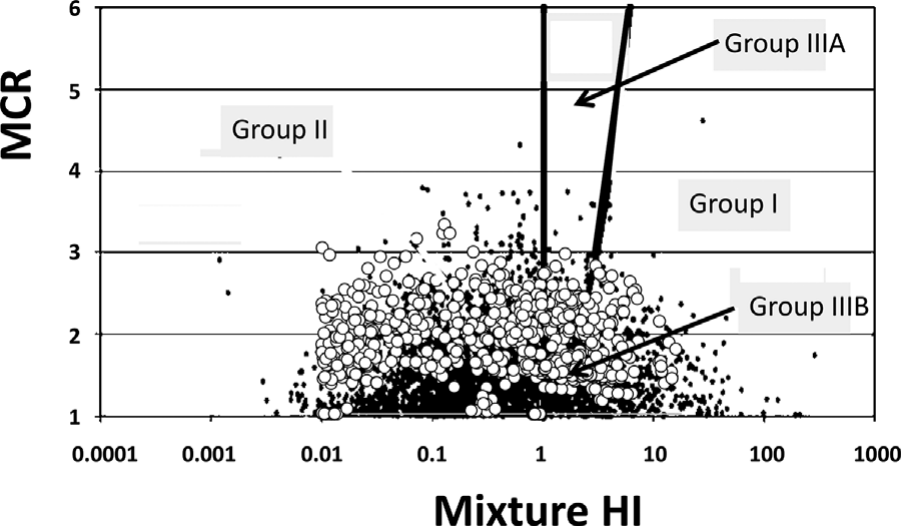
Overlay of the hazard index‐maximum cumulative ratio plots of 4380 measured concentrations of plant protection products in US watersheds (Vallotton and Price 2016) and of 7246 daily samples with associated predicted environmental concentrations from the agriculture subcatchment. Hazard indices were based on acute aquatic benchmarks for ecotoxicological effects and on the 5th percentiles of the species sensitivity distribution no‐observed‐effect concentrations, respectively, with the latter representing a more sensitive endpoint. Black dots indicate field data; white dots indicate current model results. HI = hazard index; MCR = maximum cumulative ratio.

A striking feature of the results was that the finding that the PEC variability resembled the observed ranges of the respective measured concentrations in river water samples (EMPODAT), despite considerable variation of modeled and measured data and technical limits regarding measuring compounds in field samples (Figure 3). The most striking observation was that the in silico modeled land‐use scenarios (Figures 4 and 5) yielded an HI‐MCR plot similar to that from a field study in which 12 to 81 PPPs were measured per sample (Vallotton and Price 2016; Figure 7), although the field study employed acute risk benchmarks (while we applied chronic ones). The difference between the present study and the agriculture study (Holmes et al. 2018) is caused by the use of regulatory acceptable concentrations to define mixture HIs in that study (this includes affected fractions of 100–1000 across compounds). Comparisons between predicted and observed data suggest that many of the findings of the present study can occur in true catchments. Therefore, the key patterns (below) bear relevant insights for assessing and managing complex mixtures in relation to land use.

### Key patterns in the data

The similarities of exposures and hazard plots allow for some key observations.

First, land use matters. Land use appears to imply a typical chemical signature in receiving water bodies. A signature consists of a typical chemical composition (chemicals, concentrations) and exposure time aspects (durations, frequencies). Attempts to solve existing mixture exposures in aquatic systems could therefore focus on decoupling land use from aquatic systems, for example, via buffer zones, wastewater treatment, or reduced urban runoff emission events. Such actions would imply a change in emission of suites of chemicals, with those suites including the set of chemicals of high priority within the land use. Abating chemical risks can utilize a suite of options, not solely a chemo‐centric approach (National Research Council 2009; Munthe et al. 2017); and it was, for example, shown earlier that an analysis of spatial associations between emission points and water bodies with sensitive functions (drinking water production, protected nature) can be a basis to reduce impacts via smart spatial arrangements (Coppens et al. 2015) and that clever strategies may be utilized to reduce adverse effects of chemicals and other water quality parameters (Malaj et al. 2014; Barclay et al. 2016). From upstream to downstream, land‐use influences on smaller tributaries may be characterized by mixtures with greater exposures and simpler composition, with a “land‐use dilution” effect in the downstream direction (López‐Serna et al. 2012).

Second, flow and runoff events matter, related to rain events. Even though it was expected that domestic emissions would result in relatively constant exposures, the opposite is true in the smaller tributaries in our case. The results highlight the importance of rain events and subsequent dilution phenomena. Smaller rivers may be characterized by high temporal variability in chemical concentrations, whether or not there is a constant or an intermittent emission source (domestic vis à vis agriculture spraying/runoff and urban runoff runoff). Species in flowing aquatic systems can thus be exposed to mixtures that change rapidly in composition. A recent example (König et al. 2017) showed large changes of MECs of untreated wastewater emissions in the Danube over a scale of a few kilometers only. Note that the PECs predicted for the subcatchments (current model) in reality could imply higher exposures at the points where true chemical emissions occur (e.g., edge‐of‐field exposures for agricultural chemicals and end‐of‐pipe exposures at wastewater‐treatment plant outlets and sewer overflows). The spatial and temporal variation we modeled implies challenges for the design of monitoring schemes for flowing waters and indicates that spatiotemporal variation may disturb a straightforward interpretation of MEC data vis à vis the regulatory standards such as AA‐EQS (Holt et al. 2000). For example, there may be doubts whether the MECs of a set of water samples are “representative” for the system, given spatiotemporal variability that may be an order(s) of magnitude. Modeling can help to improve understanding of the mixture risks of such systems.

Third, the choice of the assessment benchmarks matters. The integrated scenario analyses differ in this respect from the individual scenario studies (Holmes et al. 2018; Diamond et al. 2018; de Zwart et al. 2018), where various toxicity standards were used (see also Table 1). The uniform use of AA‐EQS values in the present study resulted in a large number of days triggering regulatory concern, whereas inspection of the ecological implications of direct effects of mixture exposures (chronic or acute) showed substantially lower fractions of samples potentially causing direct effects on species assemblages (related to both peak exposures as well as nonchronic exposure times). This difference shows that it is important not to overinterpret criteria exceedances, such as the PNEC or the AA‐EQS. The exceedance of such a criterion triggers regulatory concern, which should be translated into more specific information on the potential occurrence of direct ecological effects, secondary poisoning effects, or human health concern or into a trigger to improve the EQS itself when the affected fraction for one or more compounds is high. Avoiding misinterpretations has been proven useful for water quality management (Henning‐de Jong et al. 2009).

Fourth, prioritization choices matter. Prioritization helps in selecting of cost‐effective abatement strategies. A suite of prioritization options can be envisaged, and these result in vastly different lists of compounds for further attention (Guillén et al. 2012). The present study shows the effects of prioritization choices. Relevant information can be obtained from comparing land uses (clear ranking), exposure types (chronic or intermittent), and chemicals within mixtures. The latter is often used in practice, relating to the current identification of priority hazardous substances and substances prioritized for adoption on a “watch list” (regulatory attention triggered [European Commission 2013]). The observation of land use–specific chemical signatures suggests that chemicals that rank high in priority may serve as surrogates of co‐occurring, nonmodeled, or measured substances (Bradley et al. 2017). Regulatory priority substances may be indeed prioritized but may also be of marginal importance for a catchment. Of the modeled compounds cypermethrin is a priority substance for European water policies and ethinyl estradiol is identified as a candidate for the watch list (European Commission 2013). In the present study, we found various substances ranking high in various ways which are not prioritized or watch list chemicals in the context of current regulation (European Commission 2013), for example, deltamethrin, permethrin, bifenthrin, galaxolide, sulfomethoxazole, caffeine, carbamazepine, pendimethalin, flufenacet, mesosulfuron‐methyl, and fluoxastrobin. Regulatory attention may be warranted beyond regulatory lists, in line with other categorization options (Götz et al. 2010). River basin management is likely served best by a critical application of prioritizations, looking at land use, temporal aspects, chemicals of generic interest (e.g., at the European scale), and chemicals of interest given land‐use practices. For a subcatchment, listed priority compounds may pose negligible risks within a given catchment and, conversely, nonlisted compounds may be of high local priority for management. Neglect of compounds because of absence from a central listing can be called a case of unjustified reification. Reification is the process through which concepts (such as “priority compounds”) are increasingly interpreted as facts. Reification fallacies may seriously affect policy making (Bradbury 1989; Hyman 2010). Unjustified interpretations can induce type I errors (risk signals triggering abatement costs, without the signal being related to true impacts [Prato et al. 2014]) as well as type II errors (the potential impacts of many chemicals and their mixtures are neglected or remain unknown because of limitations of current science).

Fifth, the analyses always resulted in a clear identification of some chemicals contributing most to risks in mixtures. This phenomenon seems to be universal in field‐related mixture studies, as substantiated by a variety of other assessments (Zijp et al. 2014; Vallotton and Price 2016; Backhaus and Karlsson 2014; Gustavsson et al 2017; de Zwart 2005; Harbers et al. 2006; see also Figure 7). The outputs of the present study suggest strong simplifying patterns of risk in highly diverse sets of mixture exposures. Land use–related chemical signatures appear to exist, whereby mixture effects are commonly caused by a few chemicals (for a given toxicological endpoint), although those few chemicals differ with land use and time (Munz et al. 2017).

Sixth, the reporting of findings as chemical footprint information summarizes the data for an area in easily understood metrics: the multiplication factor that mixture toxic stress of a sample exceeds a benchmark, which can be interpreted as a measure of the number of times a sample needs to be diluted before the effects are below the benchmark. In this evaluation, the dilution factors needed for the different land‐use scenarios were 6, 19, and 9 for agriculture (realistic winter wheat scenario), a city (10 000 people/10 km^2^), and a mixed–land use scenario (Figure 1) to yield 95% of the species protected against NOEC exceedance because of mixture exposure for 95% of the days. Note that, commonly, various fate processes that we did not model may lower exposures in field conditions, which likely results in lower risks and chemical footprints. The predicted chemical footprint values are in line with other chemical footprint analyses for Europe (Zijp et al. 2014; Bjørn et al. 2014). In addition, the change in chemical footprint can be determined for varying catchment configurations (of urban runoff+domestic, agriculture, nature), and the effects of abatement options on the footprint can be explored (Table 4). Such summaries enable exploratory investigations as to the ecological risk reduction of altering landscape structure or impacts of alternative chemicals used for specific goals (e.g., choice of PPPs) or of chemical‐specific or generically effective abatement strategies, such as buffer zones (Van Wezel et al. 2017).

### Further analyses

Further data analyses are possible, for example, investigating which taxonomic groups are likely to be most affected by mixtures, checking time‐weighted averaged exposures and the effects of the rainfall threshold causing city runoff, and analyses based on measured efficacies of, for instance, buffer zones between human activities and water systems. The refinement for taxonomic groups was already worked out in detail for the water samples of MCR group IIIB of the agriculture scenario (Holmes et al. 2018). Such analyses can refine insights into potentially sensitive groups. Because this effect is most prominent for the agriculture scenario and the original scenario study presents such outcomes in detail, we refer to that study for details of this kind (Holmes et al. 2018).

## CONCLUSIONS

Based on the conceptual and practical evaluation of an integrative scenario, blending earlier reviewed agriculture, domestic, and urban runoff scenario data and acknowledging the limitations of this purely in silico study, we conclude the following. 1) It is possible to create a catchment‐oriented approach, encompassing land use–related emissions of chemicals, rain events, and hydrological phenomena, to predict likely chemical profiles in receiving river catchments: the PECs generated by this approach bear a reasonable relationship with measured concentrations of chemicals and the predicted patterns of ecological risks, regarding both their magnitude as well as their maximum cumulative ratios, bear a reasonable resemblance to the pattern based on field data. 2) The land use–based approach, with realistic rain events and flow variation, results in highly variable mixture compositions in space and time (composition and concentrations of chemical mixtures) but also in simplified signatures and prioritizations. 3) The outcomes demonstrated spatiotemporal variability of exposure and potential ecological impacts of chemical mixtures in human‐dominated systems but also allowed for simplifying generalizations, such as the potential for various meaningful prioritizations for risk management. 4) The complexity of true catchments and land uses can be addressed through science‐based approaches that consider exposure scenarios for a wide range of ecosystems and land‐use types (in the present study dominated by agricultural, urban, and domestic wastewater‐treatment inputs), but this requires developing “road map“ scenarios with typical exposures for prospective and retrospective risk assessments and linking to management actions. 5) The varying exposure patterns can be described across ecosystem and land‐use types by converting loadings to environmental concentrations in time‐varying river flows and finally ecotoxicologically relevant endpoints, such as HQs and HIs and mixture toxic pressures, that can be related in a tiered way to expected net mixture impacts. 6) The explanation of outcomes of modeled or measured water quality assessments requires specific attention, to avoid overinterpretation of lower‐tier methods. 7) The proposed approach for evaluating chemical mixture risks has a wide range of potential regulatory applications where approaches to mixture risk assessment are needed.

## Supplemental Data

The Supplemental Data are available on the Wiley Online Library at DOI: 10.1002/etc.3960.

## Disclaimer

The opinions expressed in the present study are those of the authors and not their respective employers.

## Data availability

Data, associated metadata, and calculation tools are available from the corresponding author (Leo.Posthuma@rivm.nl).

## Data Accessibility

## Supporting information

This article includes online‐only Supplemental Data.

Supporting Data S1.

## References

[etc3960-bib-0001] Backhaus T , Karlsson M. 2014 Screening level mixture risk assessment of pharmaceuticals in STP effluents. Water Res 49:157–165. 24321250 10.1016/j.watres.2013.11.005

[etc3960-bib-0002] Barclay JR , Tripp H , Bellucci CJ , Warner G , Helton AM. 2016 Do waterbody classifications predict water quality? J Environ Manage 183:1–12. 27621038 10.1016/j.jenvman.2016.08.071

[etc3960-bib-0003] Bjørn A , Diamond M , Birkved M , Hauschild MZ. 2014 Chemical footprint method for improved communication of freshwater ecotoxicity impacts in the context of ecological limits. Environ Sci Technol 48:13253–13262. 25347848 10.1021/es503797d

[etc3960-bib-0004] Brack W , Dulio V , Ågerstrand M , Allan I , Altenburger R , Brinkmann M , Bunke D , Burgess RM , Cousins I , Escher BI , Hernández FJ , Hewitt LM , Hilscherová K , Hollender J , Hollert H , Kase R , Klauer B , Lindim C , Herráez DL , Miège C , Munthe J , O'Toole S , Posthuma L , Rüdel H , Schäfer RB , Sengl M , Smedes F , van de Meent D , van den Brink PJ , van Gils J , van Wezel AP , Vethaak AD , Vermeirssen E , von der Ohe PC , Vrana B. 2017 Towards the review of the European Union Water Framework management of chemical contamination in European surface water resources. Sci Total Environ 576:720–737. 27810758 10.1016/j.scitotenv.2016.10.104 PMC8281610

[etc3960-bib-0005] Bradbury JA. 1989 The policy implications of differing concepts of risk. Science, Technology, & Human Values 14:380–399.

[etc3960-bib-0006] Bradley PM , Journey CA , Romanok KM , Barber LB , Buxton HT , Foreman WT , Furlong ET , Glassmeyer ST , Hladik ML , Iwanowicz LR , Jones DK , Kolpin DW , Kuivila KM , Loftin KA , Mills MA , Meyer MT , Orlando JL , Reilly TJ , Smalling KL , Villeneuve DL. 2017 Expanded target‐chemical analysis reveals extensive mixed‐organic‐contaminant exposure in U.S. streams. Environ Sci Technol 51:4792–4802. 28401767 10.1021/acs.est.7b00012 PMC5695041

[etc3960-bib-0007] Conley JM , Evans N , Cardon MC , Rosenblum L , Iwanowicz LR , Hartig PC , Schenck KM , Bradley PM , Wilson VS. 2017 Occurrence and in vitro bioactivity of estrogen, androgen, and glucocorticoid compounds in a nationwide screen of United States stream waters. Environ Sci Technol 51:4781–4791. 28401766 10.1021/acs.est.6b06515 PMC11247474

[etc3960-bib-0008] Coppens LJC , van Gils JAG , ter Laak TL , Raterman BW , van Wezel AP. 2015 Towards spatially smart abatement of human pharmaceuticals in surface waters: Defining impact of sewage treatment plants on susceptible functions. Water Res 81:356–365. 26102555 10.1016/j.watres.2015.05.061

[etc3960-bib-0009] de Zwart D. 2005 Ecological effects of pesticide use in The Netherlands: Modeled and observed effects in the field ditch. Integr Environ Assess Manag 1:123–134. 16639894 10.1897/ieam_2004-015.1

[etc3960-bib-0010] de Zwart D , Adams W , Galay Burgos M , Hollender J , Junghans M , Merrington G , Muir D , Parkerton T , De Schamphelaere KAC , Whale G , Williams R . 2018 Aquatic exposures of chemical mixtures in urban environments: Approaches to impact assessment. Environ Toxicol Chem 37:703–714 (*this issue*). 28861906 10.1002/etc.3975

[etc3960-bib-0011] de Zwart D , Posthuma L. 2005 Complex mixture toxicity for single and multiple species: Proposed methodologies. Environ Toxicol Chem 24:2665–2676. 16268170 10.1897/04-639r.1

[etc3960-bib-0012] Diamond J , Altenburger R , Coors A , Dyer SD , Focazio M , Koelmans AA , Leung KMY , Servos MR , Snape J , Tolls J , Zhang X. 2018 Use of prospective and retrospective risk assessment methods that simplify chemical mixtures associated with treated domestic wastewater discharges. Environ Toxicol Chem 37:690–702 (*this issue*). DOI: 10.1002/etc.4013. 29068498 10.1002/etc.4013

[etc3960-bib-0013] European Commission. 2013 Directive 2013/39/EU of the European Parliament and of the Council of 12 August 2013 amending Directives 2000/60/EC and 2008/105/EC as regards priority substances in the field of water policy. Official J Eur Union L226:1–17.

[etc3960-bib-0014] European Commission. 2014. Living well, within the limits of our planet. General Union Environment Action Programme to 2020. Luxembourg.

[etc3960-bib-0015] Geiser K. 2015 Chemicals Without Harm. Policies for a Sustainable World. MIT, Cambridge, MA, USA.

[etc3960-bib-0016] Gessner MO , Tlili A. 2016 Fostering integration of freshwater ecology with ecotoxicology. Freshwater Biol 61:1991–2001.

[etc3960-bib-0017] Ginebreda A , Kuzmanovic M , Guasch H , de Alda ML , López‐Doval JC , Muñoz I , Ricart M , Romaní AM , Sabater S , Barceló D. 2013 Assessment of multi‐chemical pollution in aquatic ecosystems using toxic units: Compound prioritization, mixture characterization and relationships with biological descriptors. Sci Total Environ 468–469:715–723. 10.1016/j.scitotenv.2013.08.086 24070871

[etc3960-bib-0018] Götz CW , Stamm C , Fenner K , Singer H , Schärer M , Hollender J. 2010 Targeting aquatic microcontaminants for monitoring: Exposure categorization and application to the Swiss situation. Environ Sci Pollut R 17:341–354. 10.1007/s11356-009-0167-8 19475441

[etc3960-bib-0019] Guillén D , Ginebreda A , Farré M , Darbra RM , Petrovic M , Gros M , Barceló D. 2012 Prioritization of chemicals in the aquatic environment based on risk assessment: Analytical, modeling and regulatory perspective. Sci Total Environ 440:236–252. 22809786 10.1016/j.scitotenv.2012.06.064

[etc3960-bib-0020] Gustavsson MB , Magnér J , Carney Almroth B , Eriksson MK , Sturve J , Backhaus T . 2017 Chemical monitoring of Swedish coastal waters indicates common exceedances of environmental thresholds, both for individual substances as well as their mixtures. Mar Pollut Bull 122:409–419. 28693810 10.1016/j.marpolbul.2017.06.082

[etc3960-bib-0021] Harbers JV , Huijbregts MAJ , Posthuma L , Van de Meent D. 2006 Estimating the impact of high‐production‐volume chemicals on remote ecosystems by toxic pressure calculation. Environ Sci Technol 40:1573–1580. 16568772 10.1021/es051633m

[etc3960-bib-0022] Henning‐de Jong I , Ragas AMJ , Hendriks HWM , Huijbregts MAJ , Posthuma L , Wintersen A , Jan Hendriks A . 2009 The impact of an additional ecotoxicity test on ecological quality standards. Ecotox Environ Safe 72:2037–2045. 10.1016/j.ecoenv.2009.08.009 19748120

[etc3960-bib-0023] Holmes CM , Brown CD , Hamer M , Jones R , Maltby L , Posthuma L , Silberhorn E , Teeter JS , Warne MSJ , Weltje L. 2018 Prospective aquatic risk assessment for chemical mixtures in agricultural landscapes. Environ Toxicol Chem 37:674–689 (*this issue*). 29193235 10.1002/etc.4049 PMC5873440

[etc3960-bib-0024] Holt MS , Fox K , Grießbach E , Johnsen S , Kinnunen J , Lecloux A , Murray‐Smith R , Peterson DR , Schröder R , Silvani M , ten Berge WFJ , Toy RJ , Feijtel TCM. 2000 Monitoring, modelling and environmental exposure assessment of industrial chemicals in the aquatic environment. Chemosphere 41:1799–1808. 11057621 10.1016/s0045-6535(00)00036-9

[etc3960-bib-0025] Hyman SE. 2010 The diagnosis of mental disorders: The problem of reification. Annu Rev Clin Psychol 6:155–179. 17716032 10.1146/annurev.clinpsy.3.022806.091532

[etc3960-bib-0026] Johnson AC , Donnachie RL , Sumpter JP , Jürgens MD , Moeckel C , Gloria Pereira M . 2017 An alternative approach to risk rank chemicals on the threat they pose to the aquatic environment. Sci Total Environ 599–600:1372–1381. 10.1016/j.scitotenv.2017.05.039 28531948

[etc3960-bib-0027] Kolpin DW , Furlong ET , Meyer MT , Thurman EM , Zaugg SD , Barber LB , Buxton HT. 2002 Pharmaceuticals, hormones, and other organic wastewater contaminants in U.S. streams, 1999–2000: A national reconnaissance. Environ Sci Technol 36:1202–1211. 11944670 10.1021/es011055j

[etc3960-bib-0028] König M , Escher BI , Neale PA , Krauss M , Hilscherová K , Novák J , Teodorović I , Schulze T , Seidensticker S , Kamal Hashmi MA , Ahlheim J , Brack W . 2017 Impact of untreated wastewater on a major European river evaluated with a combination of in vitro bioassays and chemical analysis. Environ Pollut 220:1220–1230. 27884472 10.1016/j.envpol.2016.11.011

[etc3960-bib-0029] Kortenkamp A , Backhaus T , Faust M. 2009. State of the art report on mixture toxicity. European Commission, Directorate General for the Environment. Luxembourg.

[etc3960-bib-0030] López‐Serna R , Petrović M , Barceló D. 2012 Occurrence and distribution of multi‐class pharmaceuticals and their active metabolites and transformation products in the Ebro River basin (NE Spain). Sci Total Environ 440:280–289. 22809787 10.1016/j.scitotenv.2012.06.027

[etc3960-bib-0031] Malaj E , von der Ohe PC , Grote M , Kühne R , Mondy CP , Usseglio‐Polatera P , Brack W , Schäfer RB. 2014 Organic chemicals jeopardize the health of freshwater ecosystems on the continental scale. Proc Natl Acad Sci USA 111:9549–9554. 24979762 10.1073/pnas.1321082111 PMC4084479

[etc3960-bib-0032] Munthe J , Brorström‐Lundén E , Rahmberg M , Posthuma L , Altenburger R , Brack W , Bunke B , Engelen G , Gawlik BM , Van Gils J , López Herráez D , Rydberg T , Slobodnik J , Van Wezel A . 2017 An expanded conceptual framework for solution‐focused management of chemical pollution in European waters. Environ Sci Eur 29:1–16. 28337403 10.1186/s12302-017-0112-2 PMC5344934

[etc3960-bib-0033] Munz NA , Burdon FJ , de Zwart D , Junghans M , Melo L , Reyes M , Schönenberger U , Singer HP , Spycher B , Hollender J , Stamm C. 2017 Pesticides drive risk of micropollutants in wastewater‐impacted streams during low flow conditions. Water Res 110:366–377. 27919541 10.1016/j.watres.2016.11.001

[etc3960-bib-0034] National Research Council. 2009 *Science and Decisions: Advancing Risk Assessment*. National Academies Press, Washington, DC, USA. 25009905

[etc3960-bib-0035] Posthuma L , de Zwart D. 2012 Predicted mixture toxic pressure relates to observed fraction of benthic macrofauna species impacted by contaminant mixtures. Environ Toxicol Chem 31:2175–2188. 22729941 10.1002/etc.1923

[etc3960-bib-0036] Posthuma L , Dyer SD , de Zwart D , Kapo K , Holmes CM , Burton GA Jr. 2016 Eco‐epidemiology of aquatic ecosystems: Separating chemicals from multiple stressors. Sci Total Environ 573:1303–1319. 27519323 10.1016/j.scitotenv.2016.06.242

[etc3960-bib-0037] Prato S , La Valle P , De Luca E , Lattanzi L , Migliore G , Morgana JG , Munari C , Nicoletti L , Izzo G , Mistri M. 2014 The “one‐out, all‐out” principle entails the risk of imposing unnecessary restoration costs: A study case in two Mediterranean coastal lakes. Mar Pollut Bull 80:30–40. 24529849 10.1016/j.marpolbul.2014.01.054

[etc3960-bib-0038] Schäfer RB , Kühn B , Malaj E , König A , Gergs R. 2016 Contribution of organic toxicants to multiple stress in river ecosystems. Freshwater Biol 61:2116–2128.

[etc3960-bib-0039] Sobek A , Bejgarn S , Ruden C , Breiholtz M. 2016 The dilemma in prioritizing chemicals for environmental analysis: Known versus unknown hazards. Environmental Science: Processes & Impacts 18:1042–1049. 27312930 10.1039/c6em90023b

[etc3960-bib-0040] Stephan CE , Mount DI , Hansen DJ , Gentile JH , Chapman GA , Brungs WA. 1985. Guidelines for deriving numerical national water quality criteria for the protection of aquatic organisms and their uses. PB 85‐227049. US Environmental Protection Agency, Duluth MN.

[etc3960-bib-0041] US Environmental Protection Agency. 2009. The national study of chemical residues in lake fish tissue. Washington, DC.

[etc3960-bib-0042] Vallotton N , Price PS. 2016 Use of the maximum cumulative ratio as an approach for prioritizing aquatic coexposure to plant protection products: A case study of a large surface water monitoring database. Environ Sci Technol 50:5286–5293. 27057923 10.1021/acs.est.5b06267

[etc3960-bib-0043] Van Straalen NM , Denneman CAJ. 1989 Ecotoxicological evaluation of soil quality criteria. Ecotox Environ Safe 18:241–251. 10.1016/0147-6513(89)90018-3 2693071

[etc3960-bib-0044] Van Wezel A , Ter Laak T , Fischer A , Bauerlein P , Munthe J , Posthuma L . 2017 Mitigation options for chemicals of emerging concern in surface waters: Operationalising solutions‐focused risk assessment. Environmental Science: Water Research & Technology. DOI: 10.1039C7EW00077D.

[etc3960-bib-0045] Zijp MC , Posthuma L , Van de Meent D. 2014 Definition and applications of a versatile chemical pollution footprint methodology. Environ Sci Technol 48:10588–10597. 25111657 10.1021/es500629f

