# Supplementary material for: Prospective mixture risk assessment and management prioritizations for river catchments with diverse land uses
Source: Environ Toxicol Chem. 2018 Feb 12;37(3):715–28. doi: 10.1002/etc.3960 (PMC5873277; doi:10.1002/etc.3960)
Supplement: Supplementary file 1 — Supporting Data S1. [file ETC-37-715-s001.docx]

SUPPORTING INFORMATION

PROSPECTIVE mixture risk assessment AND MANAGEMENT PRIORITIZATIONS FOR RIVER CATCHMENTS WITH DIVERSE LAND USES

Leo Posthuma^||,§*^, Colin Brown^‡‡^, Dick De Zwart^‡^, Jerry Diamond^≡^, Scott D. Dyer^#^, Christopher M. Holmes^†^, Stuart Marshall^⊕^, G. Allen Burton, Jr.^◊^

Running Head:

Blending mixture exposure scenarios for risk assessment

Addresses and affiliations

^||^ RIVM, Centre for Sustainability, Environment and Health, P.O. Box 1, 3720 BA Bilthoven, the Netherlands. E: [Leo.Posthuma@rivm.nl,](mailto:Leo.Posthuma@rivm.nl,) T:+31-30-2742295

^§^ Radboud University, Department of Environmental Science, Institute for Wetland and Water Research, Faculty of Science, Radboud University, Nijmegen, The Netherlands

^‡‡^ University of York, Environment Department, Heslington, York, YO10 5DD, UK

^‡^ Mermayde, Groet, the Netherlands

^≡^ Tetra Tech, Owings Mills, USA

^#^ The Procter and Gamble Company, Cincinnati, USA

^†^ Waterborne Environmental, Inc., Leesburg, Virginia, USA

^⊕^ Unilever, Safety and Environmental Assurance Centre, Unilever, Sharnbrook, Bedford, United Kingdom (retired)

^◊^ University of Michigan, Ann Arbor, U.S.A.

* Corresponding author. To whom correspondence may be addressed

# M&M: Integrating land use scenarios

## Land uses, geography and hydrology

Three separate chemical emission scenarios (Agriculture-AGR, Domestic-DOM, Urban-URB) along with no-chemical emission scenario (Nature-NAT) were developed on the basis of literature reviews and by combining hydrological- and ecotoxicological modelling techniques with various regulatory judgment criteria [1-3]. DOM and URB relate to the land use CITY. The scenarios were substantiated in a spreadsheet model for a catchment of 100 km^2^, which can consist of one to ten sub-catchments of 10 km^2^ each, linked in a river network. The spreadsheet is an expansion of the model described for the AGR-scenario [1]. Details are in that study. The characteristics that are relevant for the current study are described below.

In the spreadsheet, land use combinations can be varied by assigning the land uses to sub-catchments in a spatial arrangement. The position (p) of a sub-catchment relative to the main (100-km^2^) catchment outlet was defined by the number of days travel to the outlet (i.e., “lag” time), where p=0 (sub-catchment containing the main catchment outlet), p=-1 (sub-catchment one day from the catchment outlet), p=-2 (sub-catchment two days from the catchment outlet, see Figure 1 of the main paper).

The model contains baseline flow information for each sub-catchment land use type to represent the flow arising from realistic conditions relating to the selected soil characteristics, land area, water retention time, and river stretch dimensions. The flow data are dynamic, changing with rainfall events, and flow statistics can be summarized for the bottom of each sub-catchment, and be aggregated to the net outflow of the whole catchment.

## Hydrology

The modeling framework for hydrology is based on the catchment model SWAT (<http://swat.tamu.edu/>), a model that is used to predict the effect of management decisions on water, sediment, nutrient and pesticide yields with reasonable accuracy on large, ungauged river basins. The inputs of surface runoff in this model are consistent with the model PRZM (<https://www.epa.gov/pesticide-science-and-assessing-pesticide-risks/models-pesticide-risk-assessment>) with the Soil Conservation Services (SCS) Curve Number approach, which was used in the AGR scenario paper. SWAT scales up beyond the 1-ha field simulated for the AGR-scenario and it incorporates the effects of baseflow and runoff in urban areas.

SWAT was used to generate separate simulations for the three types of 10-km^2^ sub-catchments: natural areas (land uses with no significant application of chemicals by man and therefore no significant emissions), agriculture, and city. The model was run for one soil type, taken from the EFSA FOCUS model (R1 scenario) and with curve numbers adjusted for natural, agricultural or urban land use.

Flow simulations were generated for the 20-year period (7246 days, March 1, 1975 to December 31, 1994) using the realistic rainfall data from the AGR scenario analyses [1]. A spreadsheet was used to combine the 10 km^2^ outputs (daily loadings and flow) to ensure consistency with the loading approach, transparency, and scenario evaluations (e.g. impact of different proportions of each land use, or impact of an urban area being located at the top or bottom of the catchment).

The 20-year time period was modelled on a per-day basis, with historic rain events and associated flows. In addition to the hydrological model, the following details define the selected conditions in the model. The original AGR scenario calculations are characterized by a dynamic hydrology for a small, field-edge water body with emissions driven by pesticide applications (drift) or storm events (runoff). For this scenario (UK-winter wheat), daily loadings were obtained from the outputs of AGR-FOCUS runs (using PRZM and a spray drift calculator). Resulting values are relevant to 1-ha of wheat, combining spray drift and surface runoff loadings. These outputs were linearly scaled for the current study to loadings relative to the 10-km^2^ sub-catchment using the proportion of wheat in the sub-catchment; for the illustrative simulations undertaken here, it was assumed that the entire 10-km^2^ sub-catchment (i.e. 100% of the surface area) was cropped with winter wheat and treated with the pesticide program described in the AGR scenario analysis [1]. The original DOM-scenario generates constant concentrations in a large river defined to deliver a 10-fold dilution of concentrations leaving the wastewater treatment plant. For the integrated DOM-scenario, the loadings were determined for a population estimate for a 10-km^2^ sub-catchment of urban/suburban area of 10,000. The loadings (mass) were back calculated from effluent-PECs based on effluent flow. Thereupon, the effluent loadings were scaled to the 10-km^2^ population. The original URB-scenario was driven by information on chemical monitoring data (chemical identities and concentrations), while the frequency of contaminant emissions and scale of the receiving water body were not defined. For the integration of the URB-scenario in the current study, the aim was to obtain daily loadings at the base of the 10-km^2^ sub-catchment. The 20-year rainfall data were used to determine the days when loadings occur due to stormwater runoff. Therefore, the daily rainfall amount on each 10-km^2^ sub-catchment was examined, and a threshold was applied to determine whether urban runoff was triggered.

## Land uses, chemical emissions and their assessment

### General design and approach

The original agriculture scenario (UK winter wheat scenario, [1]) was used as basis for the catchment scenario spreadsheet model. This scenario considers twenty consecutive years, with realistic weather conditions (rain), the hydrological effects of which were modelled on a day-to-day basis for the sub-catchment flows. Here, concentrations of chemicals were calculated at sub-catchment outlets as derived from daily loadings and flow for each type of sub-catchment, followed by dynamic calculation of the mixture concentrations at the bottom of the sub-catchments and the total catchment configuration. The resulting Predicted Environmental Concentrations at the (sub-)catchment outlet were judged via various methods, which were in part similar to the original scenario studies on AGR, DOM and URB, but also in part different. Some judgment criteria are specific to individual compound groups (such as e.g. the Regulatory Accepted Concentration, RAC, for edge-of-field risk assessment of plant protection products). Additional evaluation criteria were selected so as to allow for comparison across land use scenarios on a consistent basis.

### Agriculture

Compounds. The selected agricultural land use scenario AGR is the original row crops scenario (winter wheat, U.K., [1]). The AGR scenario considers the typically used winter wheat plant protection product applications (substances, timings of use, amounts of use), applied according to defined agricultural practices. The study considers 13 active substances, applied annually over the 20 years, with application rates derived from actual use statistics. Emissions to water (loads) were those used in the original study.

Emissions. The FOCUS R1 runoff scenario was used in the original study to model chemical emissions. Cited from the detailed Supporting Information of that study [1]: “[It] comprised a free-draining light silt soil with small organic matter content (5% sand, 82% silt, 13% clay, 1.2% organic carbon) coupled with a temperate climate with moderate precipitation (600-800 mm annual average rainfall, 100-200 mm annual rainfall, 6.6-10 ^o^C average temperature in autumn and spring), gently to moderately sloping land (2-4%) and a range of crop types including winter cereals that was simulated for the current purpose. Related to environmental fate parameters, half-lives for degradation in soil were selected from aerobic laboratory studies undertaken at 20^o^C and normalized to soil moisture content at pF2 (FOCUS 2000). The soil-water partition coefficients normalized to soil organic carbon content (Koc) and Freundlich exponent (nf) were selected from standard batch studies. Where the number of studies available exceeded four, standard regulatory practice was followed with the geometric mean of all available values as input to the model for degradation half-life and Koc and the arithmetic mean used for nf (FOCUS 2014). Generally, there were two natural water/sediment values available and here the arithmetic mean was taken for degradation in the water and sediment phases.” For the current study, the scenarios were based on 100% of AGR-land grown with treated winter wheat, and PECs at the edge-of-field was assumed to be equal to the PECs at the outlet of the sub-catchment (sorption/degradation during transport from point of emission to sub-catchment outlet is ignored, and travel times to the sub-catchment outlet are assumed to be <1day).

Evaluating risk. The judgment of PECs in the present study was made with various methods, harmonized amongst the land use scenarios, and (partly) different from the approaches in the original AGR scenario study. The methods are described in the main paper, and details are in Section S.I. 2. For the original AGR-study on winter wheat, risks were evaluated using Regulatory Acceptable Concentrations (RACs), evaluated at the edge-of-field scale water body. RACs are effects assessment endpoints expressed in terms of a permissible concentration in the environment that is directly used in the risk assessment by comparing it to the (predicted) aquatic exposure concentration. If the concentration is lower than the RAC, the environmental effects of a PPP are assumed to be acceptable and consequently the risk is deemed low. In the original study, RACs were also calculated for taxonomic groups (i.e. fish, invertebrates and primary producers), using the available data and the methodology appropriate for an EU risk assessment of PPPs, applying the assessment factors according to the EFSA Aquatic Guidance (related to quantity and quality of available ecotoxicity data). Higher tier ecotoxicity data were also used, using the endpoints generally as presented in the respective EU assessments and following current EFSA guidance. The outcomes of the original AGR-study were based on various approaches, amongst which the RAC and cumulated RAC-ratios using the lowest value from all the taxonomic groups as Tier-1 approach, and taxonomic-group specific RACs as Tier-2 approach. The present study considers concentrations at the outlet of the AGR-sub-catchment, or at the outlet of the whole catchment of the scenario model run.

### Domestic

Compounds. The original domestic scenario [2] considered nutrients, consumer products, pharmaceuticals, hormones, solvents and specialty chemicals. Emission sources were diverse, and the current study focuses on domestic emissions only. The emissions are considered to occur via point sources, and are related to population density and characterized by a continuous discharge, treated on a pathway including a waste water treatment plant (type modelled for the integration scenario: activated sludge). In the domestic scenario, typical household chemicals were selected for the exposure and risk modelling of the integration paper (with relatively high contribution to risks in the original scenario [2]).

Emissions. Domestic loadings were modelled using the following characteristics: 200 L of water use per day per capita, 10,000 people inhabiting the sub-catchment of 10 km^2^, a WWTP-dilution factor of 10, a basic river flow of 0.231 m^3^/sec, and loading- and WWTP-removal efficiencies (activated sludge technique) as in S.I. Table 1. The loadings combined with the hydrological model yielded the PECs at the sub-catchment outlet. Note that the loadings are modelled as constant (per-capita constant use of household chemicals), while the hydrological dynamics result in time- and flow-dynamic PECs at the outlet.

| **Full name** | **Abbreviation used** | **Per capita use rate (g/cap/d)** | **Removal (Activated Sludge)** | **Effluent Mass Loading (g/d)** |
| --- | --- | --- | --- | --- |
| 1-OH-Benzotriazole | D-BTZ | 0.003 | 0.0% | 30 |
| Acesulfame | D-ACS | 0.015 | 27.0% | 109.5 |
| Benzalkonium chloride | D-BAC | 0.0082 | 99.8% | 0.1632 |
| Caffeine | D-CAF | 0.30 | 84.0% | 480 |
| Carbamazepin | D-CMZ | 0.0014 | 22.0% | 10.92 |
| Erythromycin Sulfomethoxazole | D-SMX | 0.005 | 58.0% | 21 |
| Ethinylestradiol | D-EE2 | 0.0000018 | 82.0% | 0.0032 |
| HHCB (Galaxolide) | D-HHCB | 0.02 | 56.0% | 87.12 |
| Ibuprofen | D-IBU | 0.019 | 90.0% | 18.55 |
| LAS | D-LAS | 0.60 | 99.0% | 60 |
| Methylisothiazolinone | D-MI | 0.0006 | 50.0% | 3 |
| TiO | D-TiO | 0.098 | 97.0% | 29.25 |
| Zinc acetate | D-ZnA | 0.0032 | 74.0% | 8.29 |
| ZnO | D-ZnO | 0.065 | 74.0% | 169 |

S.I. Table 1. Details on the modelled data for selected chemicals from the domestic scenario (D=domestic).

Evaluating risk. The judgment of PECs in this study was made with various methods, harmonized amongst the land use scenarios, and (partly) different from the approaches in the original scenario studies. The methods are described in the main paper, and details are in Section S.I. 2. The Tier 1 assessment of PECs in the original study was based on predicted no effect concentrations (PNEC). These were derived as threshold values following the EU guidance [4] by applying assessment factors to the reported effect data and selecting the lowest value of the three species groups (fish, aquatic plant, and crustaceans) as the PNEC. The Tier 2 assessment of PECs in the original study consisted of the comparison of the chronic no effect concentrations of separate species groups, which were used as the threshold values. If chronic values were not available, acute values were used and divided by a factor of ten to extrapolate from acute to chronic effects. We acknowledge that for some chemicals, acute to chronic ratios may be much higher based on endocrine disruption effects. The Tier 2 differentiation between species groups provides enhanced diagnostic resolution and may allow identification of the species group(s) at greatest risk. It was noted that for ZnO, the Tier 1 threshold values reflect the toxicity of the Zn^2+^ ion, which is considered to cause the toxic effects. The Tier 2 threshold values for ZnO, and TiO are based on tests with nanomaterials, which are the actual ingredients of the sunscreen products. Risk Characterization Ratios (RCR) in the original study were computed based on PECs and the PNEC values obtained as described above. The RCRs for individual chemicals were then summed, providing a cumulative RCR (EC, 2003) as a measure for approximating the risk of the mixture for each treatment type, although it is acknowledged that this may imply aggregating very different PNEC-types. In the Tier 2 approach of the original study, a cumulative RCR was computed for three organism groups – fish, daphnia, and algae – separately. The aggregated values were named ‘hazard quotients’ (being cumulative RCRs) and were determined for each organism group. It was noted that the chronic toxicity values collected in Tier 2 were not necessarily the toxicity estimates on which the PNEC of Tier 1 was based.

### Urban

Compounds. The original urban runoff scenario [3] considered PAHs, metals, and solids, and emissions of those compounds from sources such as roads, parking lots, buildings and roofs, in total 77 compounds. The available data concern defined amounts of runoff, typical event mean concentrations, defined flows in receiving waters, considering roads, buildings, parks, other structures, and hydrodynamics with intermittent exposures (rain events and run off), with industrial direct-, industrial indirect-, and domestic flowing into combined storm overflows. For the URB-modelling, a selection of the top 10 chemicals causing indications of risk were selected for the present study.

Emissions. Urban loadings were modelled in a dynamic way, but they were determined by a threshold rainfall event, which was set as the 95% percentile of the 20-year rainfall data (P95 = 10.3 mm rainfall). With an altered threshold event, the urban runoff will be more frequent with lower loads (lower threshold), or less frequent with a higher load (higher threshold). The selected threshold represents expected runoff loads and PECs as in the URB scenario paper, based on 30% land area being impervious surface connected to a drain system. In this way, a sufficient amount of rainfall in an event generates runoff and associated runoff PECs. To derive PECs from the loading events, 30% of the land area was assumed to consist of impervious surface connected to drain system, and therefore 30% of the runoff water volume was assumed to reach the water body. Data are in S.I. Table 2.

| **Full name** | **Event Mean Concentration in runoff water (g/m^3^)** | **Mass loading (g/d) for runoff volume of 3.06E+04 m^3^/day** |
| --- | --- | --- |
|  |  |  |
| Copper dissolved | 0.033 | 1.01E+03 |
| Zinc dissolved | 0.084 | 2.57E+03 |
| Deltamethrin | 0.000084 | 2.57E+00 |
| Bifenthrin | 0.00003 | 9.18E-01 |
| Benz(a)anthracene | 0.000192 | 5.88E+00 |
| Permethrin | 0.000202 | 6.18E+00 |
| Iron dissolved | 1.106 | 3.38E+04 |
| Aluminum reactive | 0.0242 | 7.41E+02 |
| Nonylphenol ethoxylate | 0.004165 | 1.27E+02 |
| Fluoranthene | 0.000887 | 2.71E+01 |

S.I. Table 2. Details on the modelled data for selected chemicals from the urban scenario for a 10.3 mm rainfall event.

Evaluating risk. The judgment of PECs in this study was made with various methods, harmonized amongst the land use scenarios, and (partly) different from the approaches in the original scenario studies. The methods are described in the main paper, and details are in Section S.I. 2. In the original study, the PECs were judged using the median EC50 of the data set of all species, and by the mixture toxic pressure, calculated via SSD_EC50_ models (msPAF_EC50_).

# M&M: Evaluating risk

## On the existence of various benchmarks

The original studies [1-3] apply various toxicity benchmarks (BMs) to judge the PECs and the mixtures at the outlets of the (sub-)catchments. The judgment criteria differed vastly across the three studies, as they were selected in the original studies in relation to the pertinent regulatory context, e.g., generic environmental assessments, or species assessments for plant protection products. In the present study we applied several benchmarks in a similar way across the land uses, to avoid repetition and to align the AGR-study to the judgment options for the other land uses and substances.

## On the benchmarks used in the present study

The specific judgment criteria in the current study were:

- For individual chemicals:
  - A regulatory benchmark concentration, AA-EQS, the Annual Average Environmental Quality Standard, as applied in the context of the European Water Framework Directive [5]
  - A scientific benchmark concentration representing the concentration at which it is predicted that 5% of the species would be affected beyond their NOEC, abbreviated as the 5^th^ percentile of an SSD_NOEC_; this value is also known as the HC5, the Hazardous Concentration for 5 percent of the species based on an SSD_NOEC_ [6], and it is interpreted often as the so-called 95%-protection level (95% of the species is protected against direct adverse effects of exposure on endpoints such as growth and reproduction)
  - A scientific benchmark concentration representing the concentration at which it is predicted that 50% of the species would be affected beyond their EC50, abbreviated as the 50th percentile of an SSD_EC50_
- For mixtures: the multi-substance toxic pressure, quantified as the chronic msPAF_NOEC_ and the acute msPAF_EC50_ in line with the models underlying the two scientific benchmarks [7], see also S.I. Section 2.4.

## On the tiers of the HI – MCR assessments in the present study

The HI-MCR plotting method [8] was applied to summarize the data of a scenario run (for a specific sub-catchment, or for the multiple land uses scenario) using different benchmark definitions, representing different tiers. A choice for another benchmark influences the position of each HI-MCR data point (modelled day) both for the HI (X-position) and the MCR (Y-position).

### Tier 1: AA-EQS

In Tier 1, the HI-MCR plots were made by defining compound-specific-HQs via regulatory protective environmental quality standards (EQSs). Generic EQSs are commonly used as regulatory criteria for water quality assessments. These EQSs are typically derived as concentrations below which negligible adverse effects occur via direct and/or secondary exposure effects on ecological structure and function, or they represent negligible effects on human health. The lowest of these critical concentrations (ecological, human health) is used as a generically protective EQS. In its derivation – to be protective also under uncertainty – a generically protective EQS is often derived by applying an additional application factor (AF) to output of weighing the available effect data. The AF serves to guarantee sufficient protection also when effect data for deriving the standard are poor, and the magnitude of the AF is thereby dependent on the amount and quality of the input data for an endpoint (higher uncertainty 🡪 higher AF 🡪 lower EQS). As different jurisdictions have different EQS-definitions, we harmonized the approach by only using EU criteria. These are the PNEC and the AA-EQS and MAC-EQS (annual-average EQS, and maximum acceptable concentration EQS). Generic PNECs and AA-EQS and MAC-EQS were obtained from RIVM’s website (<http://www.rivm.nl/rvs/>).

It should be noted that, given this way of benchmarking, the EQSs for two compounds A and B can be based on different endpoints (e.g., biodiversity protection for compound A and human health for compound B) so that the HI can be numerically quantified as the sum of the HQ-A + HQ-B*,* while this value has no meaningful biological interpretation. Moreover, due to the use of the various AFs across compounds, the value of a HQ can become (very) high (e.g., when an EQS is slightly exceeded, while it was derived with an AF of e.g. 100 or 1000). High HI-values in Tier 1 signal regulatory concern, but can – for the above reasons – be refined as to which endpoint may be affected most (ecosystems, human health) in which way (direct effects, secondary poisoning, specific endpoints), or whether there are high uncertainties contributing to the concern-signal (one or more compounds with high AF).

### Tier 2: Quantifying benchmarks for effects of mixture exposure on species assemblages

To address the weaknesses associated to the Tier 1 approach, a Tier 2 method was used in which analyses were made using ecotoxicological effect-based criteria concentrations (EBC) without AFs, similar to analyses of Malaj et al. [9].

Tier-2 values were summarized as HQ_EBC_ and HI_EBC_, with values >1 indicating that chronic or acute effects on growth and reproduction are likely within the exposed species assemblage for EBCs. This was done using EBCs based on NOECs and EC50s respectively. HI-values were derived from linear summation of HQ-values, which utilizes a linear concentration-effect model concept (per compound) and concentration addition modelling (aggregating compounds). The benchmark values themselves were derived on the basis of Species Sensitivity Distribution (SSD) models for chronic and acute endpoints from SSD_NOEC_s for chronic- and from SSD_EC50_s for acute impacts (see S.I. Section 2.4). The parameters of the underlying Species Sensitivity Distribution models (SSD_NOEC_ and SSD_EC50_) are provided in S.I. Table 3. HI_NOECS_s >1 and HI_EC50_s >1 were interpreted as indicators of direct chronic, sublethal ecological impacts of the mixtures at the (sub-)catchment outlets on species assemblages and as a species loss from exposed assemblages, respectively.

### Tier 3: Quantifying mixture toxic pressures

Third, a Tier-3 refinement was made by quantifying the chronic and acute mixture toxic pressures (msPAF_NOEC_ and msPAF_EC50_) for the 7246 daily sample days of a scenario run. In contrast to the Tier-2 approach, the msPAF-outputs are characterized by an upper limit of the predicted impact on species assemblages, i.e., at maximum, 100% of the species in an assemblage are predicted to be affected at the modelled endpoint.

## On SSD-models and mixture toxic pressures

Effect-Based Criteria (previous section) and mixture toxic pressures were calculated using Species Sensitivity Distribution (SSD) modelling, starting from EC50-based SSDs [10].

Mixture toxic pressures were calculated for all daily predicted samples at (sub-)catchment outlets. This was done using an average SSD-slope for all compounds, derived from the SSD-models for which sufficient data were available (see [11]). The mixture toxic pressure was thereupon quantified assuming concentration additivity across all compounds modelled in a land use scenario. The application of this approach is in line with the assumption utilized for the HI approach used in the Tier-1 and Tier-2 risk characterization approaches of this study, and we note that the quantitative error that might be introduced as compared to the mixed-model approach (assuming within-compound group concentration additivity, and across-compound groups response additivity) is commonly small [12]. SSD_EC50_-models were derived from downloaded ecotoxicity data. Details are in S.I. Table 3. Due to the presence of clear patterns across SSD_EC50_ and SSD_NOEC_ [11] it was also possible to extrapolate SSD_NOEC_s from the tabulated data, and to derive the mixture toxic pressures at the NOEC level. In short, the outputs of mixture toxic pressure models were derived to represent the fraction of species affected beyond their NOEC, which we characterize as experience discomfort at chronic exposure, and the fraction of species affected beyond their EC50, which is a fraction found to associate to species loss [13].


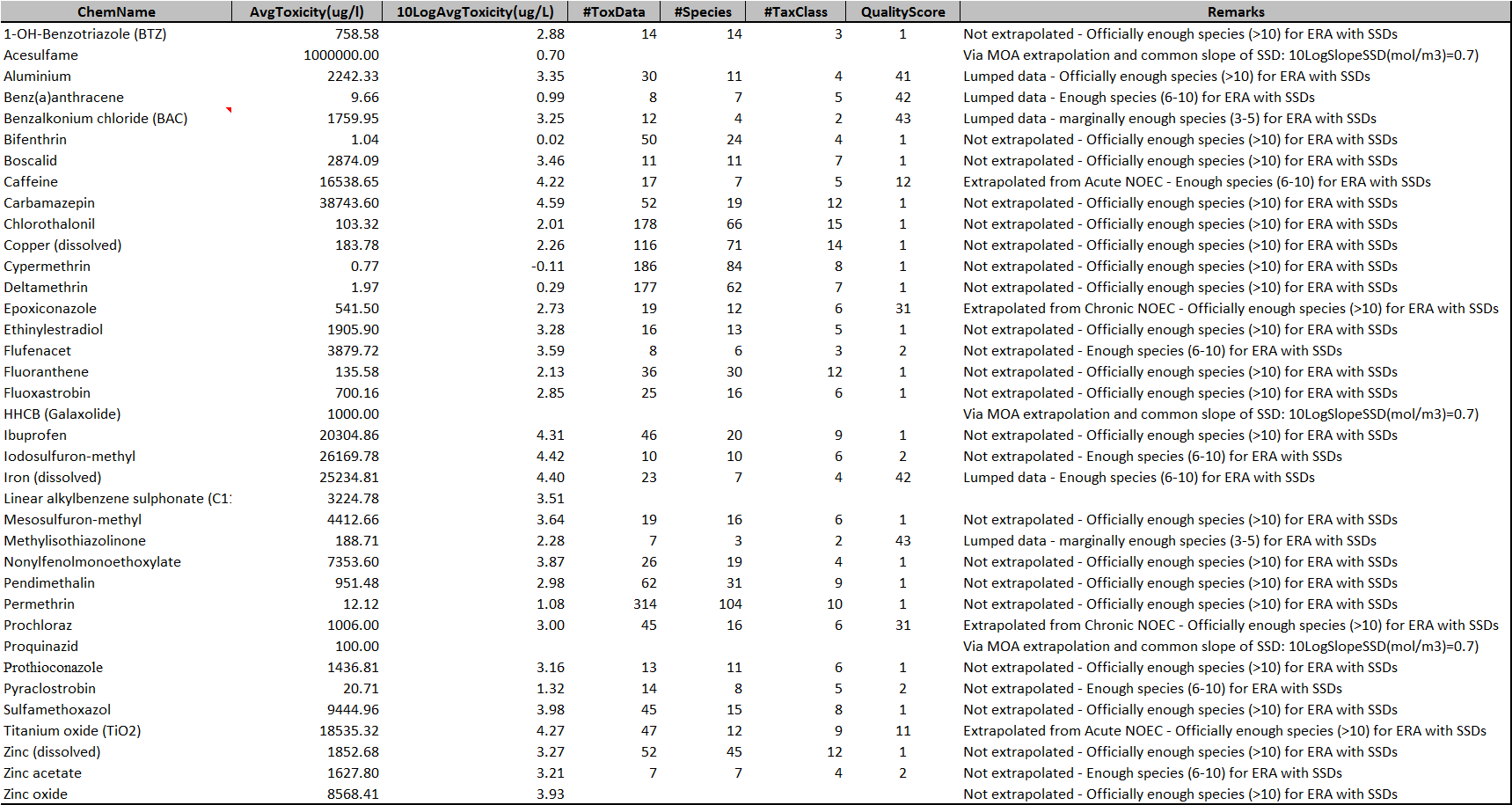


S.I. Table 3. SSD_EC50_-parameters. AvgToxicity=Average toxicity value (EC50). #ToxDat=number of input data utilized to derive the SSD. #TaxClass: number of taxa in the SSD data set. Quality score: lowest score=1 indicates a direct derivation of an SSD_EC50_ from the data. Higher quality score numbers imply the presence of (coded) extrapolation or interpolation steps, and/or less reliable fit of SSD-model to the data due to a low number of input data, see next column for explanation. E.g., “quality score 41” means that data of various types are lumped via acute-chronic extrapolation, and collated into the SSD_EC50_.

# RESULTS: Physico-chemical results

## Rainfall

Rainfall data are summarized in S.I. Figure 1, for 7246 consecutive days. Source data are rain events data associated with the FOCUS R1 exposure scenario from a European weather station, for the period of March 1, 1975 to December 31, 1994.

INSERT S.I. Figure 1 HERE


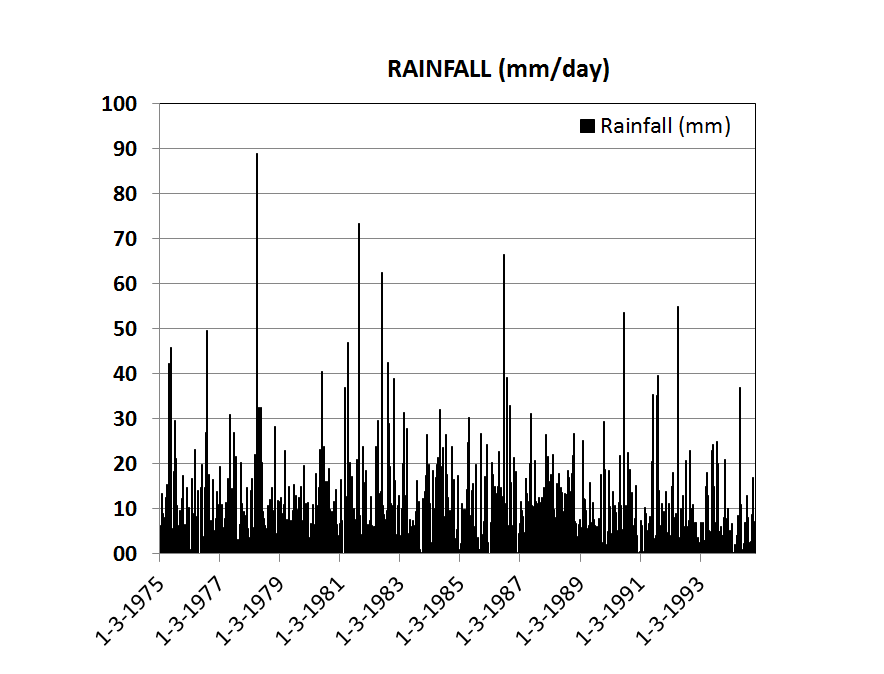

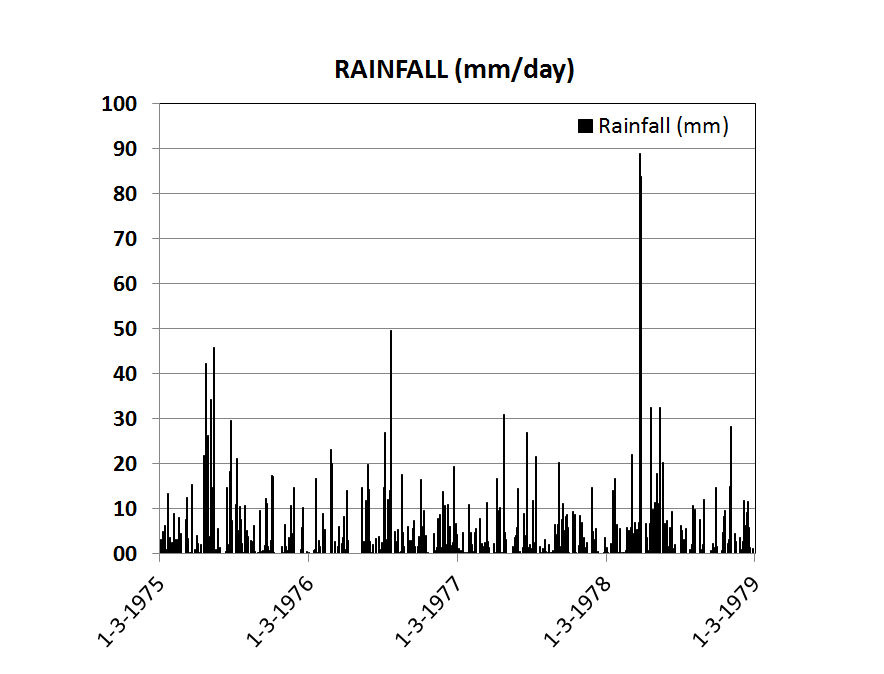


S.I. Figure 1. Rainfall. Left: for the whole period. Right: detail of rain events, for a selected period.

The natural rainfall was summarized as percentiles (P5, P25, P50, P75, P95, P99.9) for 7246 days as 0, 0, 0, 2, 10 and 53 mm.day^-1^, which implies high variability over time. This resulted in a variation of flows, and thus predicted dilutions of chemicals (see below for PECs, see SI Figures 1 and 2 for rainfall and flow details). For a CITY sub-catchment (DOM+URB), the flow percentiles were 0.03, 0.04, 0.06, 0.10, 0.35 and 1.70 m^3^.sec^-1^ (which includes the municipal wastewater effluent discharge for 10,000 people at 200 L/day (0.0231 m^3^.sec^-1^, see main paper)). For an AGR sub-catchment, the flow values were 0.005, 0.02, 0.04, 0.07, 0.29, and 1.63 m^3^.sec^-1^, which was slightly lower than for CITY. For a NAT sub-catchment, the values were 0.01, 0.03, 0.05, 0.07, 0.22, and 1.24 m^3^.sec^-1^. For the MIXED land use catchment, the values were 0.08, 0.14, 0.31, 0.51, 0.84, 2.61 and 13.32 m^3^.sec^-1^.

## Flow

Flow data are summarized in S.I. Figure 2, for 7246 consecutive days. The hydrological effects of the rainfall events differ for the different land uses, modelled here as AGR-sub-catchment and a CITY sub-catchment (with the DOM and URB scenario outputs combined along with DOM effluent), as well as for the MIXED land use for the whole catchment (see Figure 1 in main paper for MIXED catchment configuration used in this scenario).

INSERT S.I. Figure 2 HERE


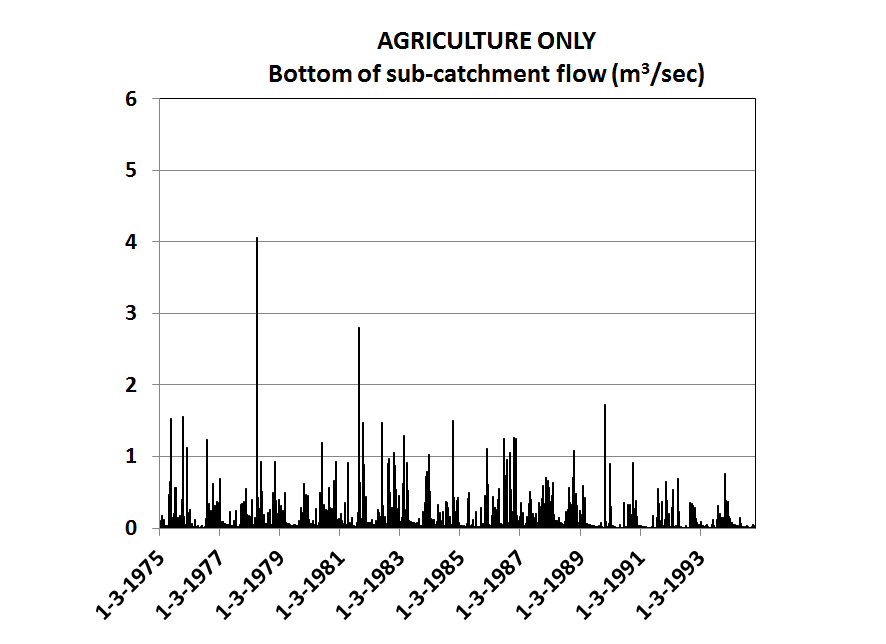

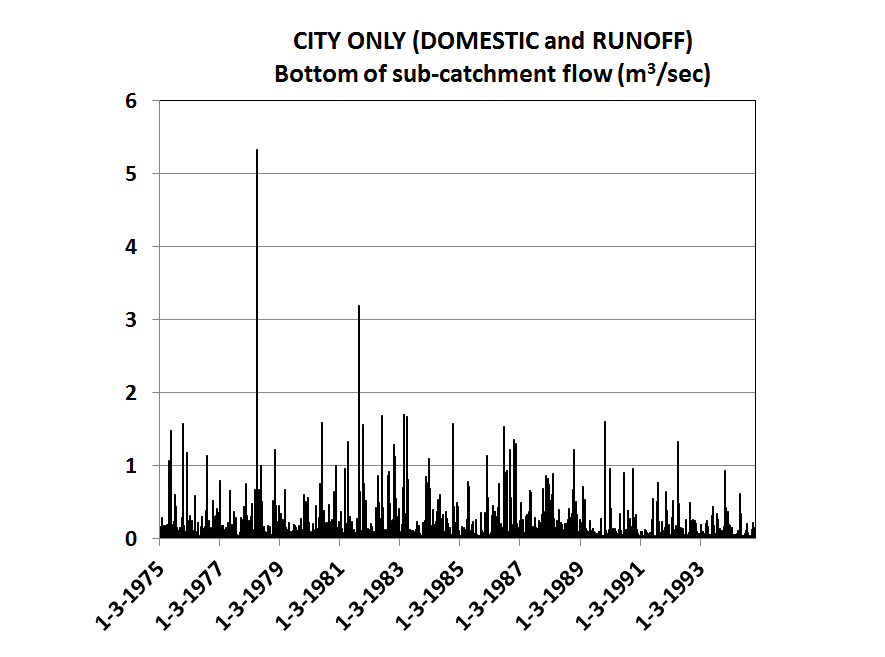

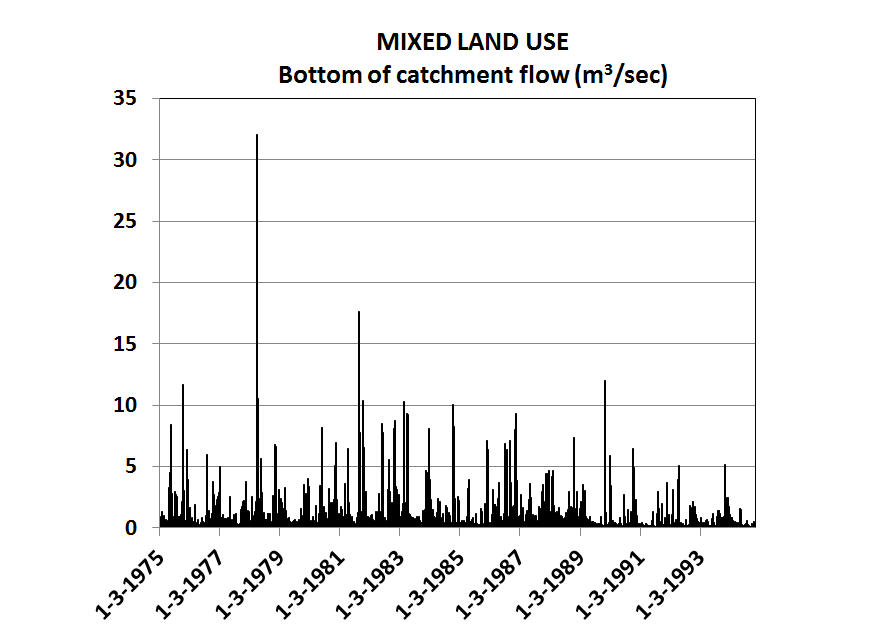


S.I. Figure 2. Illustration of flow data variability over time and across land use scenarios. Example scenarios are 10 km^2^ AGR scenario in one sub-catchment (left), 10 km^2^ CITY scenario in one sub-catchment (with DOM and URB emissions, middle), and the 100 km^2^ mixed-land use scenario of Figure 1 of the main paper. (with CITY, AGR and NAT land uses, right).

As flow variation implies chemical dilution variation, we illustrate the magnitude of the flow variability with P99.9/P5 flow ratios. High-low flow ratios were 55, 324, 128 and 94 for the scenarios CITY, AGR, NAT, and MIXED, respectively. This suggests different flow and dilution dynamics in the different sub-catchments (land uses), with large (rain- and runoff related) temporal effects superimposed on relatively lower spatial (land use) effects.

## Effluent dilution

The effluent dilution factor of the domestic wastewater treatment plant in the (sub-)catchments varied with time, and ranged from 2 to >100 for the CITY scenario (P50 ≅ 4), and from 7.5 to >100 for the MIXED land use scenario (P50 ≅ 20). As an example, the domestic discharge effluent dilution factor (DF) in the (sub-)catchment varies with time, linked to rainfall events and hydrological conditions. Domestic sub-catchment and mixed catchment DF-variation are shown in S.I. Figure 3.

INSERT S.I. Figure 3 HERE


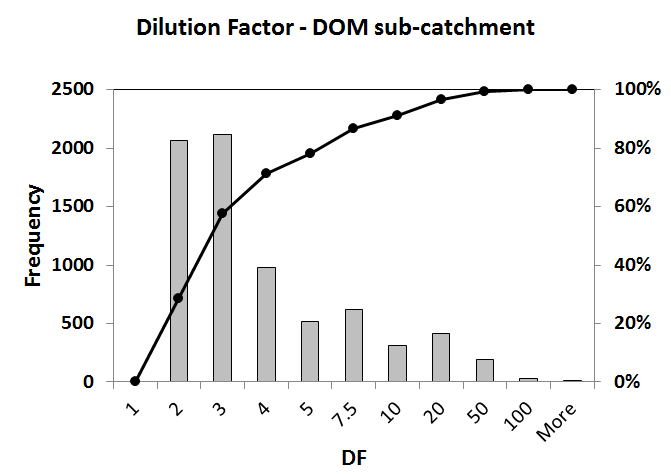

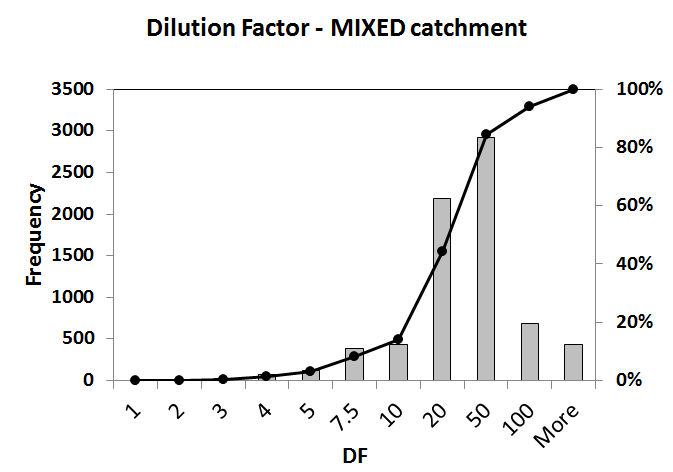


S.I. Figure 3. Variation in the dilution factor of effluents for two land use scenario layouts. Inflow of effluents from the DOM-scenario is 0.0231 m^3^/sec.

## Measured and Predicted Environmental Concentrations (MECs and PECs)

Measured and Predicted Environmental Concentrations (MECs and PECs) were collated from literature sources and the scenario modelling, respectively. MECs were obtained from the EMPODAT database, designed and managed by the NORMAN network (<http://www.norman-network.net/empodat/>; the NORMAN network is a European network of reference laboratories, research centers and related organizations for monitoring of emerging environmental substances). MECs were downloaded from EMPODAT on April 2, 2017, for all studied chemicals, by selecting “Water - Surface water – River water” as Ecosystems/Matrices query, and “Monitoring” as Type of Data Source query. Additionally MECs were obtained from the European Environment Agency Waterbase database (<http://www.eea.europa.eu/data-and-maps/data/waterbase-water-quality/>). MECs were downloaded from the Waterbase on March 31, 2017. Further MECs were obtained from other literature sources. Results are summarized in S.I. Table 4 for the chemicals studied in the AGR scenario, S.I. Table 5 for those in the DOM scenario, and S.I. Table 6 for those in the URB scenario. Note that the percentile values for EMPODAT data represent the percentiles for measured concentrations higher than the Level of Quantification (LOQ), and that – most often – measurements for a very large number of river water samples compound resulted in observed concentrations below the LOQ.

An illustration of the comparison of MECs and PECs (P95 value of the concentration at the outflow of a sub-catchment) has been made for sub-catchments with one land use (either AGR or CITY, which collates DOM and URB), and for the MIXED land use (outflow of whole catchment). Values marked grey in the MEC and PEC columns, respectively, indicate the MEC and PEC concentrations which are most closely related. For example, for the AGR scenario, a literature-based MEC of boscalid (measured in U.S. surface waters), tabulated as P50, is closely similar to the P95-PEC of the AGR sub-catchment as well as to the P95-PEC of the MIXED catchment outflow.


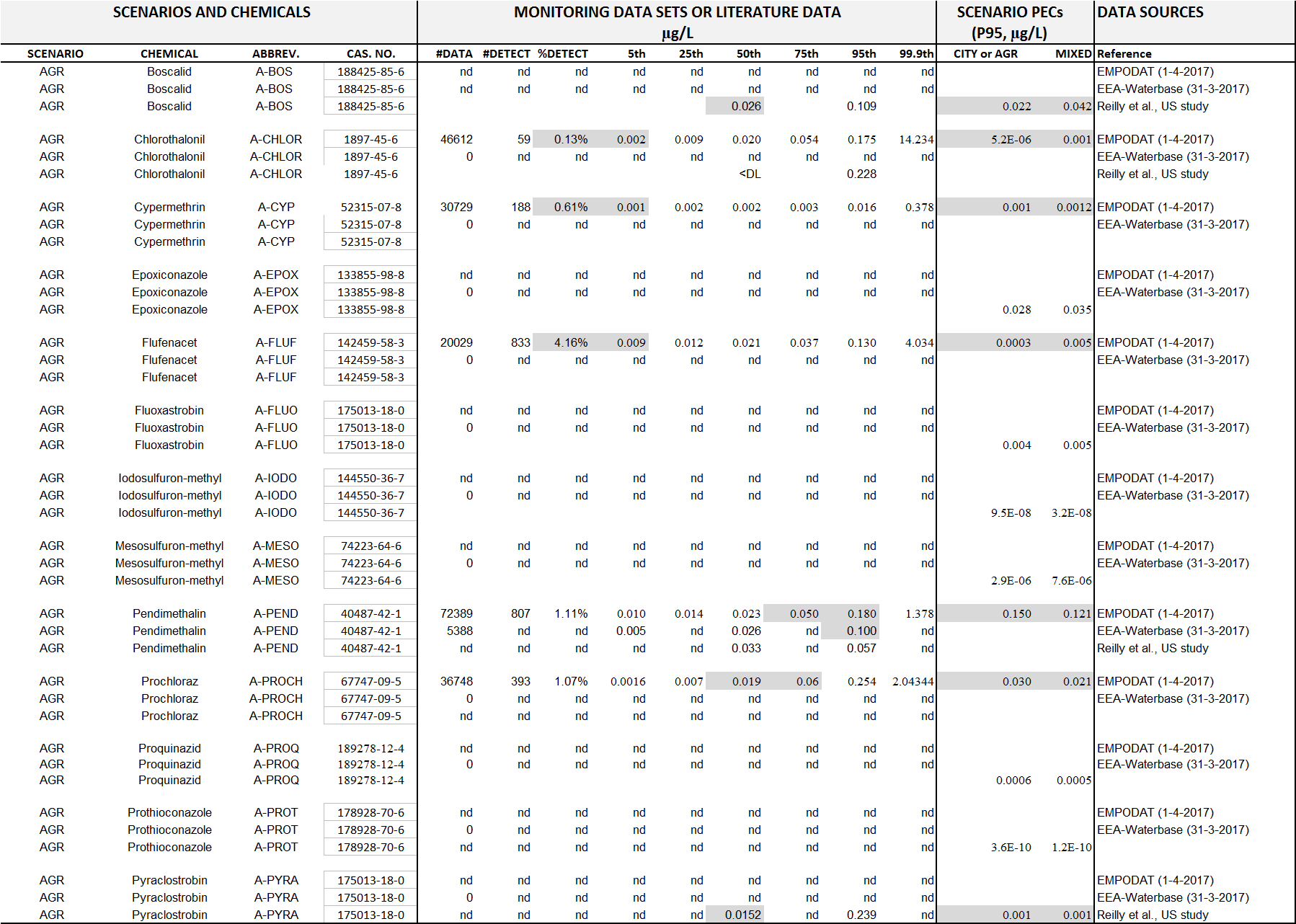


S.I. Table 4. Measured- and Predicted Environmental Concentrations of chemicals studied in the AGR scenario. Grey backgrounds indicate the closest similarity of PECs and MECs. Reilly et al. reference is: [14].


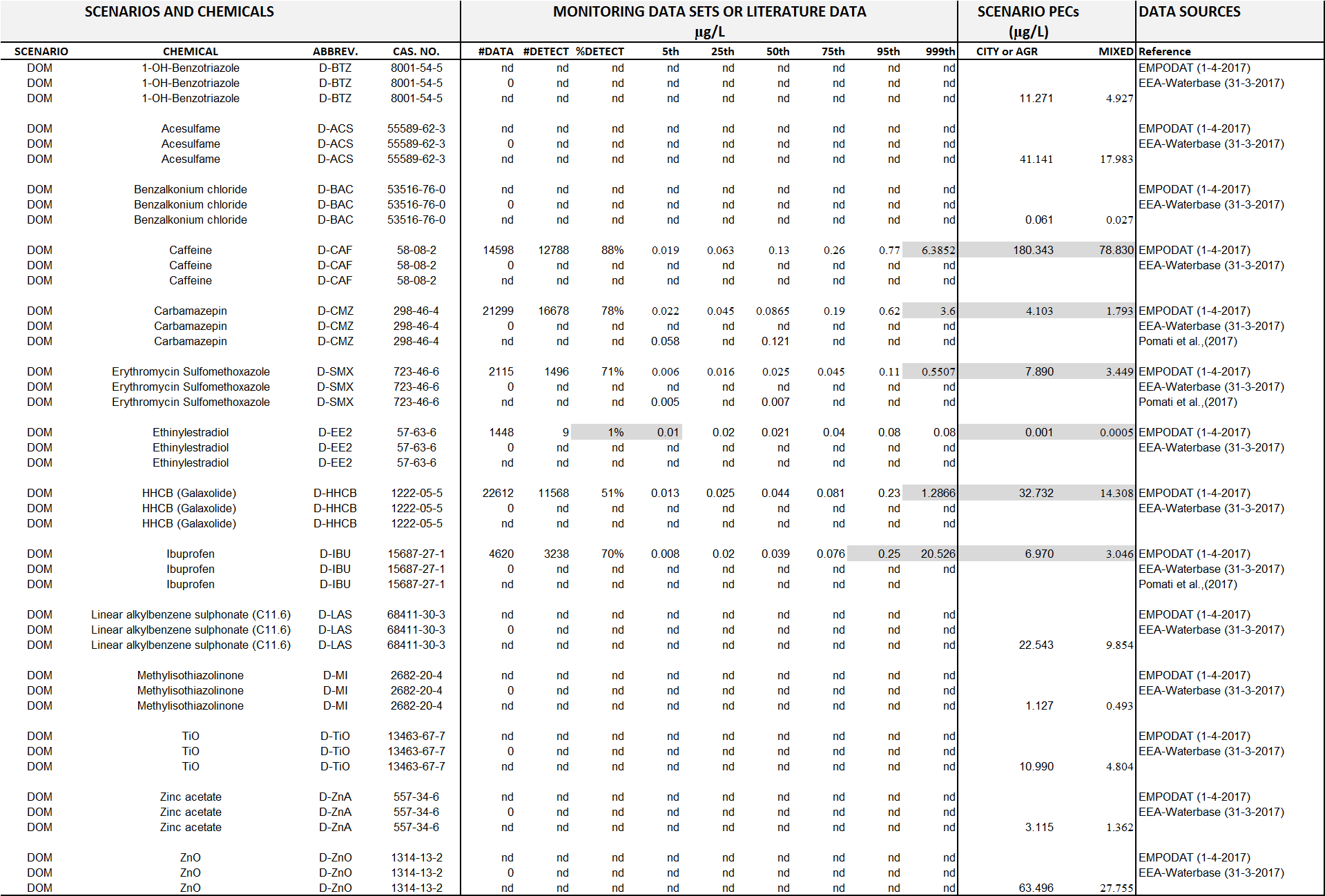


S.I. Table 5. Measured- and Predicted Environmental Concentrations of chemicals studied in the domestic scenario. Grey backgrounds indicate the closest similarity of PECs and MECs. Pomati et al reference is: [15].


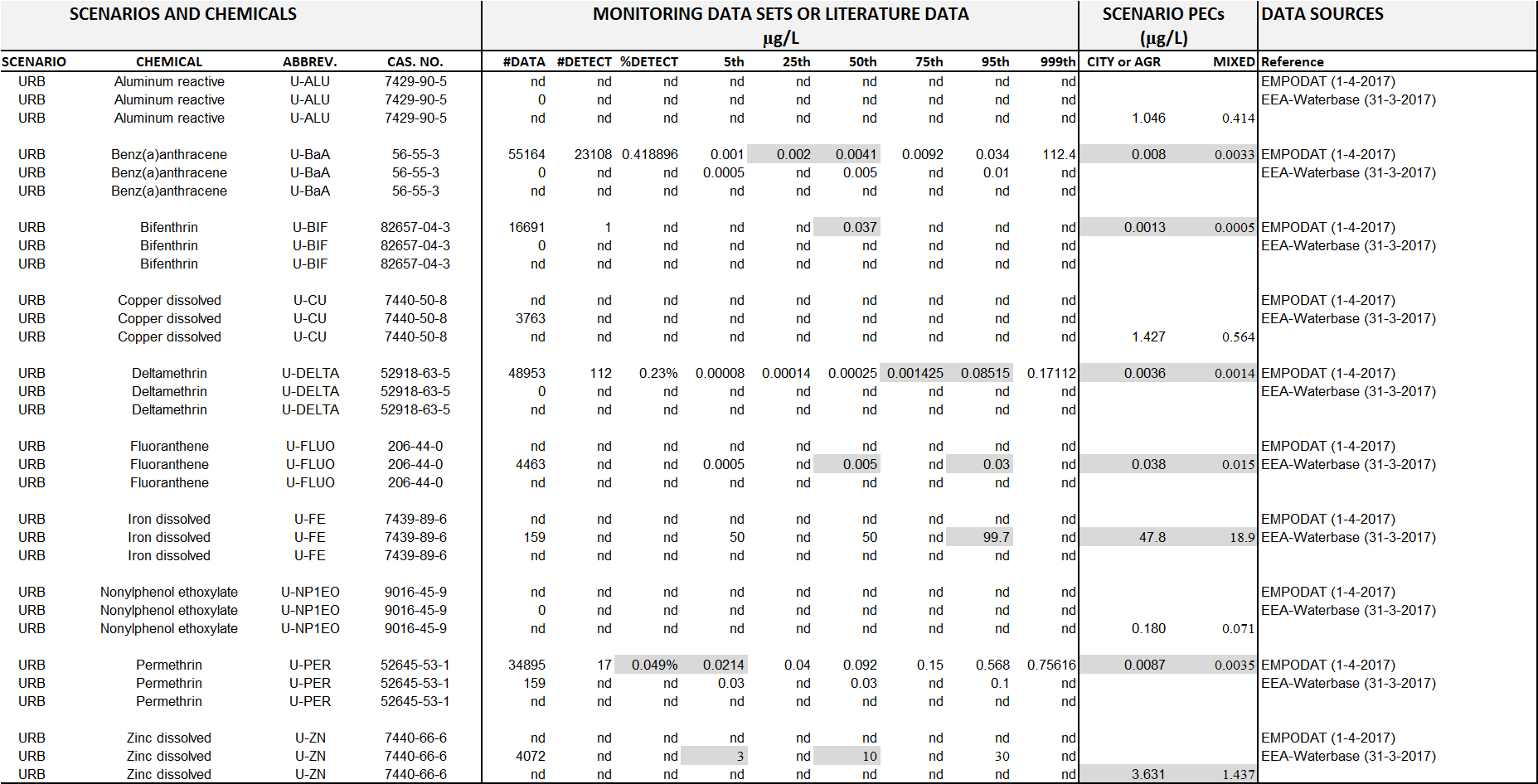


S.I. Table 6. Measured- and Predicted Environmental Concentrations of chemicals studied in the urban scenario. Grey backgrounds indicate the closest similarity of PECs and MECs.

## Predicted Environmental Concentrations (PECs) and effect endpoints

Effect endpoints were collated from the three scenario manuscripts, literature and databases. The summary data are presented in S.I. Tables 7, 8 and 9.


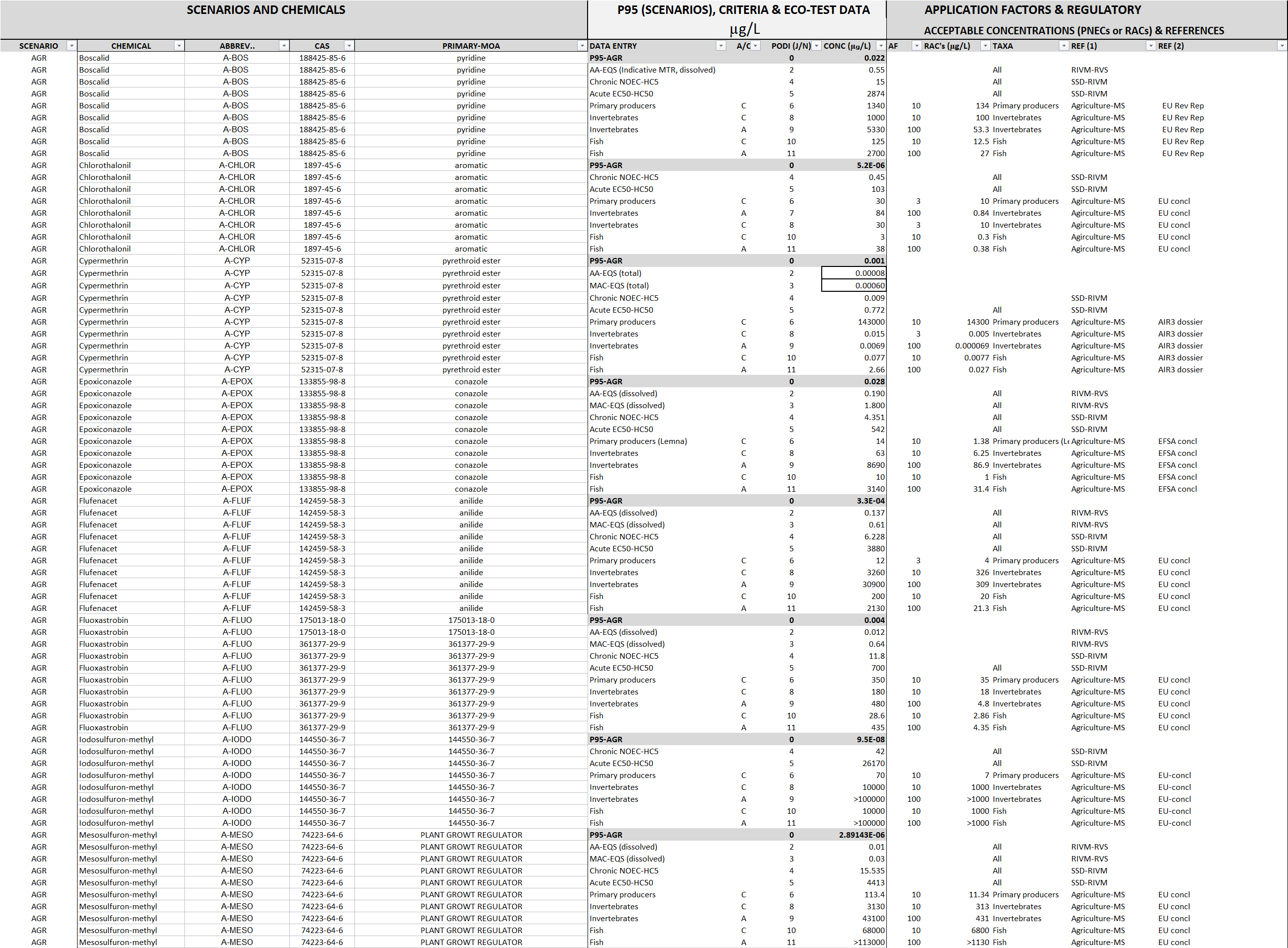


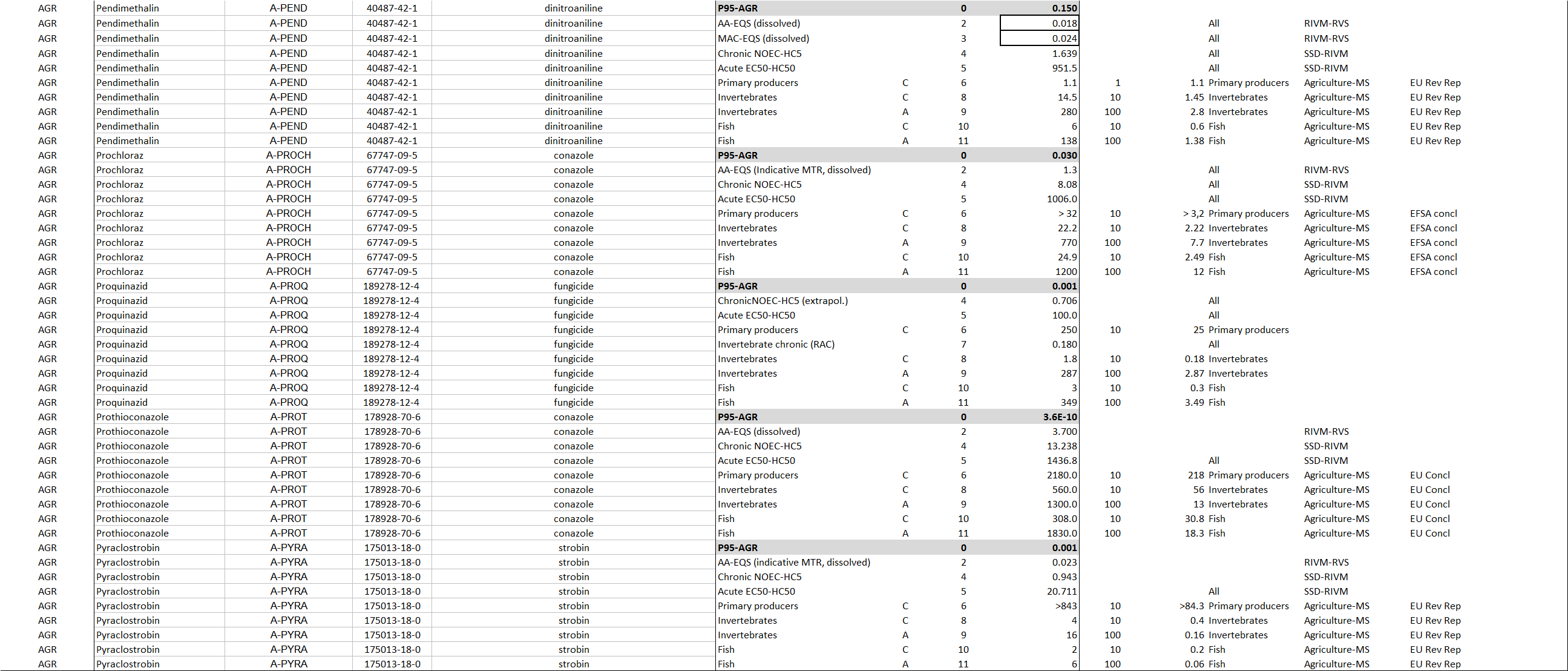


S.I. Table 7. Predicted Environmental Concentrations (P95-values at the outlet of an AGR-sub-catchment) compared with various regulatory criteria and test endpoints. Block “Scenarios and chemicals”: the chemicals, their abbreviations, their CAS-numbers, and their assigned mode of action. Block “P95 (Scenarios), Criteria & eco-test data”: the measured P95 (in grey), Acute or Chronic test endpoints (A/C), a PODI-code (Point of Departure Index, coding the different benchmarks), and the criterion or test concentration (in µg/L). Situations in which the P95-PEC at the outlet of a (sub-)catchment is higher than the regulatory criterion or test endpoint are marked with a bold outline.


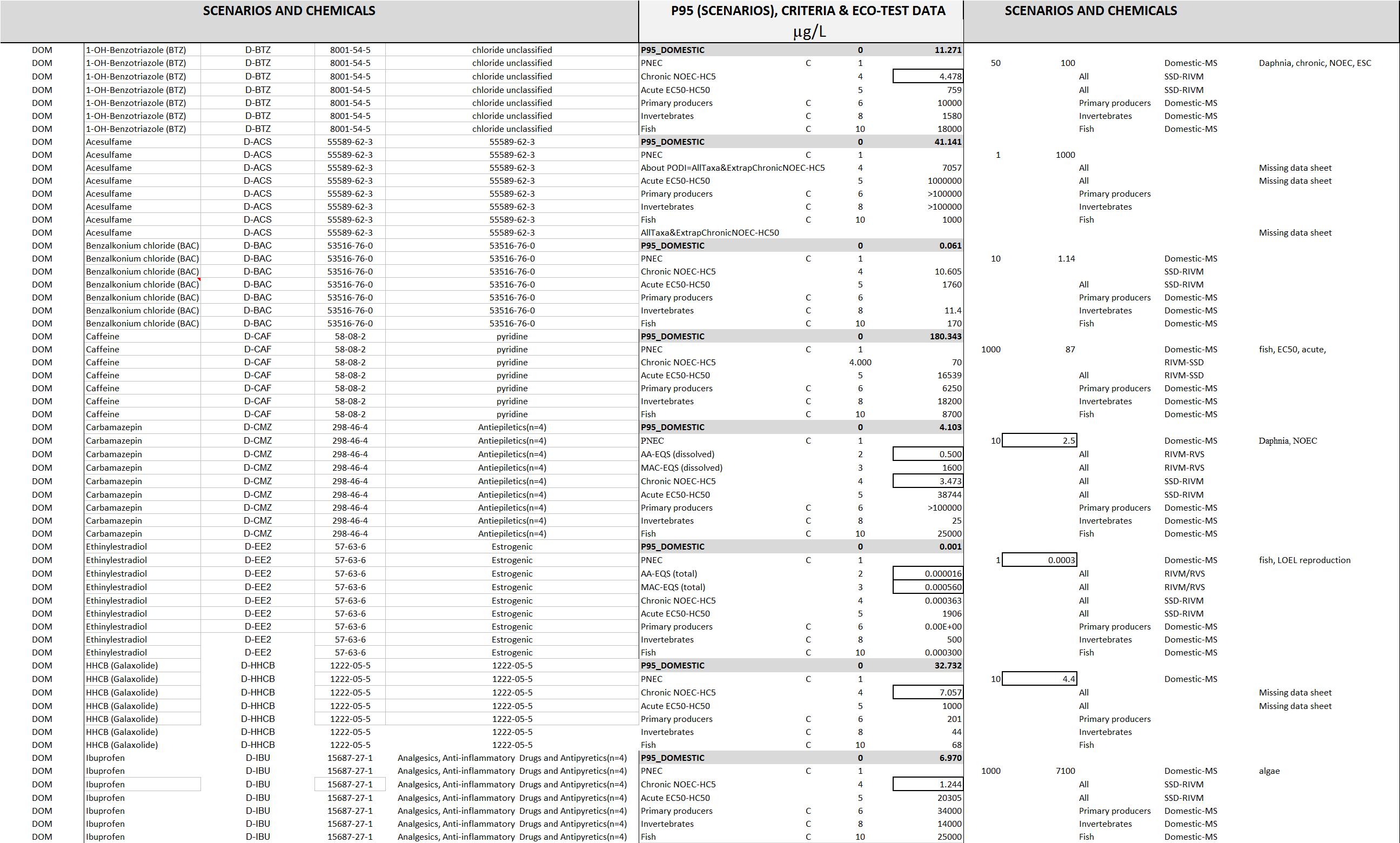


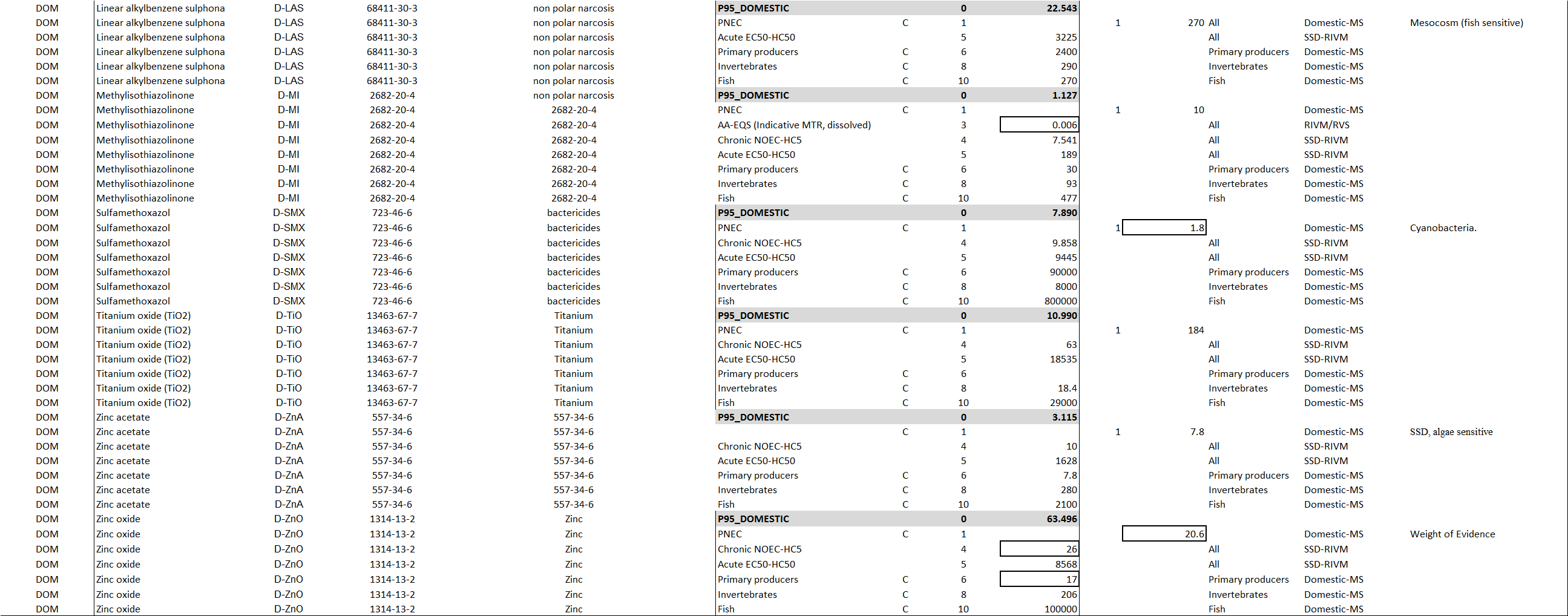


S.I. Table 8. Predicted Environmental Concentrations (P95-values at the outlet of a CITY sub-catchment, for chemicals from the DOM-scenario) compared with various regulatory criteria and test endpoints. Block “Scenarios and chemicals”: the chemicals, their abbreviations, their CAS-numbers, and their assigned mode of action. Block “P95 (Scenarios), Criteria & eco-test data”: the measured P95 (in grey), Acute or Chronic test endpoints (A/C), a PODI-code (Point of Departure Index, coding the different benchmarks), and the criterion or test concentration (in µg/L). Situations in which the P95-PEC at the outlet of a (sub-)catchment is higher than the regulatory criterion or test endpoint are marked with a bold outline.


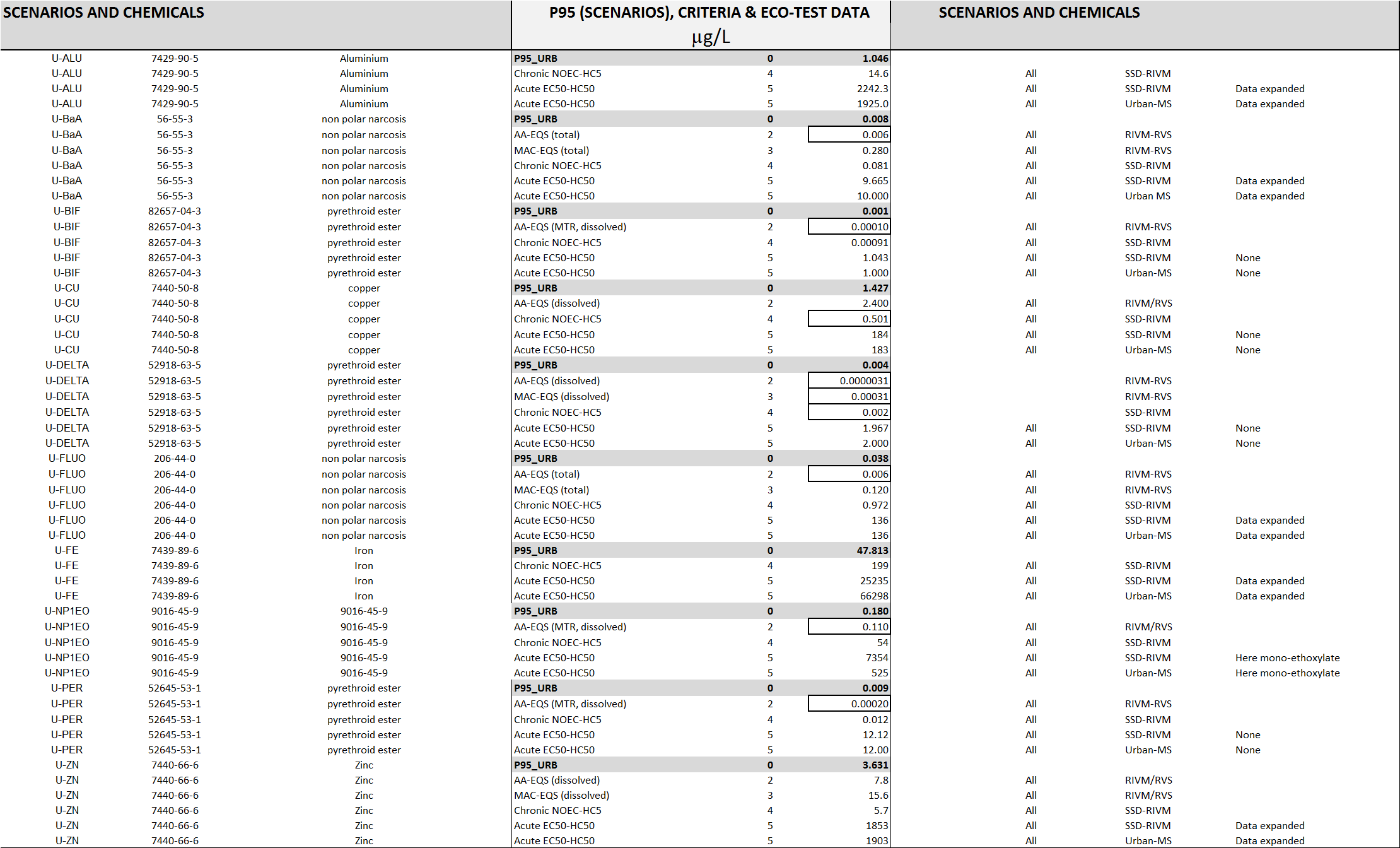


S.I. Table 9. Predicted Environmental Concentrations (P95-values at the outlet of a CITY sub-catchment, for chemicals from the URB-scenario) compared with various regulatory criteria and test endpoints. Block “Scenarios and chemicals”: the chemicals, their abbreviations, their CAS-numbers, and their assigned mode of action. Block “P95 (Scenarios), Criteria & eco-test data”: the measured P95 (in grey), Acute or Chronic test endpoints (A/C), a PODI-code (Point of Departure Index, coding the different benchmarks), and the criterion or test concentration (in µg/L). Situations in which the P95-PEC at the outlet of a (sub-)catchment is higher than the regulatory criterion or test endpoint are marked with a bold outline.

# RESULTS: Prioritizations

A prioritization of sample predictions regarding aspects of exposure time is summarized in S.I. Table 10.


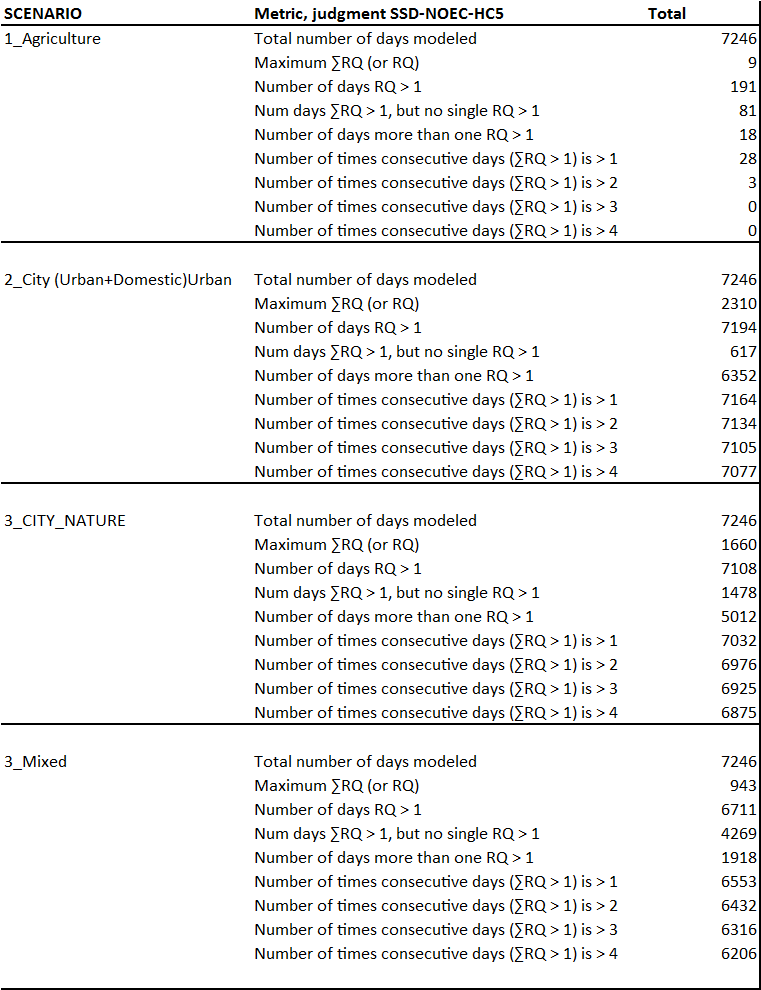


S.I. Table 10. Prioritization information regarding maximum HI and exposure time variables.

A prioritization of the relative role of chemicals in the scenarios is summarized in S.I. Table 11. In the two sub-tables that are shown, a prioritization is made for the AGR-scenario chemicals. The evaluation is made for (left) exceedances of the regulatory AA-EQS (or the comparable type concentration value used for this) and (right) for the exceedance of the 95^th^-protection level. In the regulatory evaluation, the maximum HI_AA-EQS_ was 450 for one of the modelled days, and a number of 714 days for which the mixture hazard index exceeded 1. Cypermethrin was contributing most to the AA-EQS hazard indices (79%), followed by pendimethalin (17%). The next most important compounds regarding relative contributions to the HI are mesosulfuron-methyl, flufenacet and fluoxastrobin, etc. The prioritization for the aspect of time shows a different ranking. For example, an exceedance of the HQ for fluoxastrobin (using the AA-EQS as criterion) was predicted for 34% of the days at which the HI exceeded 1.

In the other table (right), the ranking towards magnitude and number of days is made using the 95^th^ protection criterion (SSD-NOEC-HC5) as criterion to define the concentration above which direct chronic ecotoxicological effects are expected. The values for the maximum mixture HI and the number of days for which the mixture HI>1 are much lower, relating to the multiple endpoints targeted for protection via the AA-EQS and the application factors, relating to uncertainties, in the AA-EQS assessment. Not only the magnitude of peak exceedances and numbers of days of exceedances is lowered, but also the rank order of chemicals contributing to these exceedances changed. This table ranks chlorothalonil high in its contribution to the maximum HI, and cypermethrin as compound ranking high in its relative number of days contributing to mixtures exceedances >1.


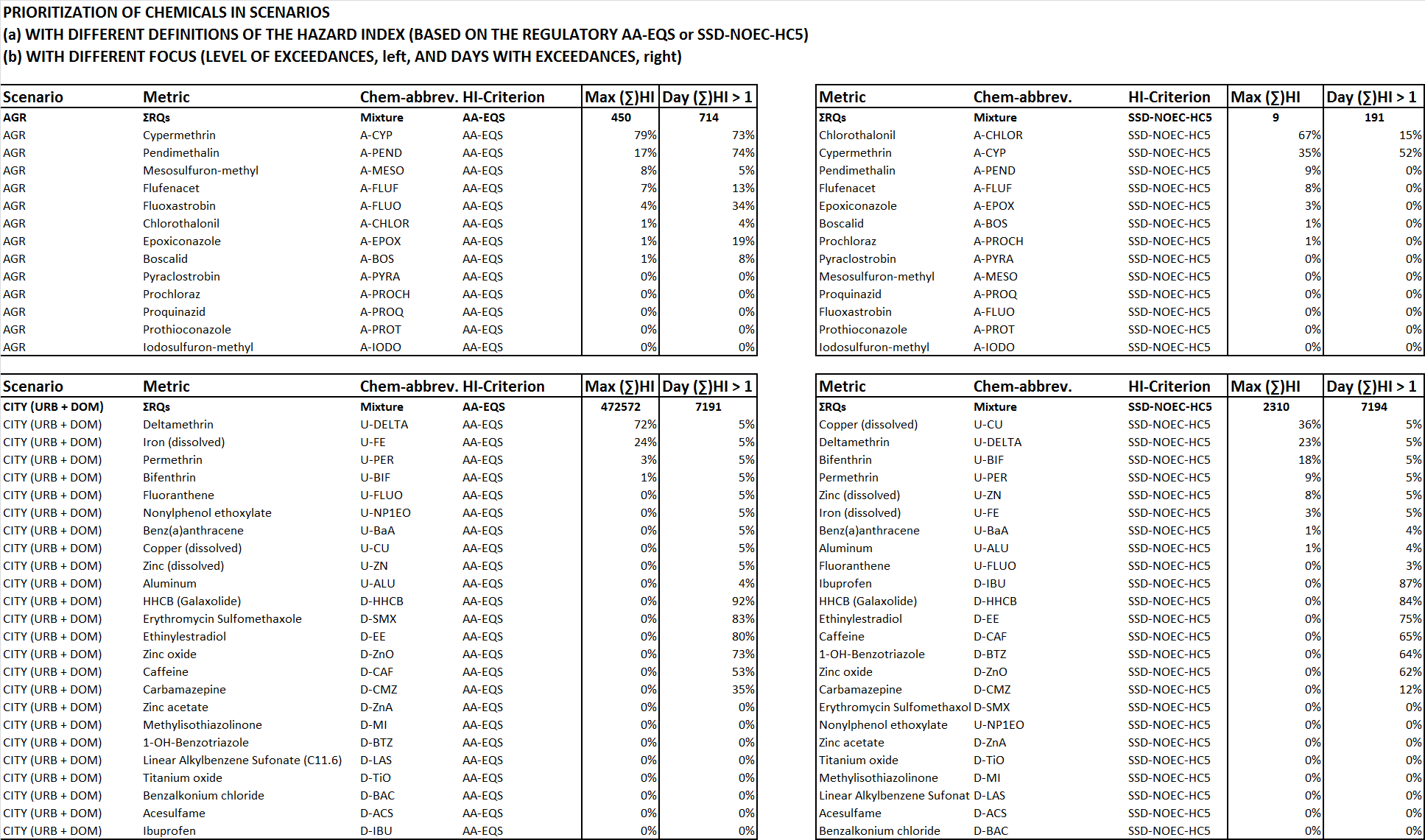


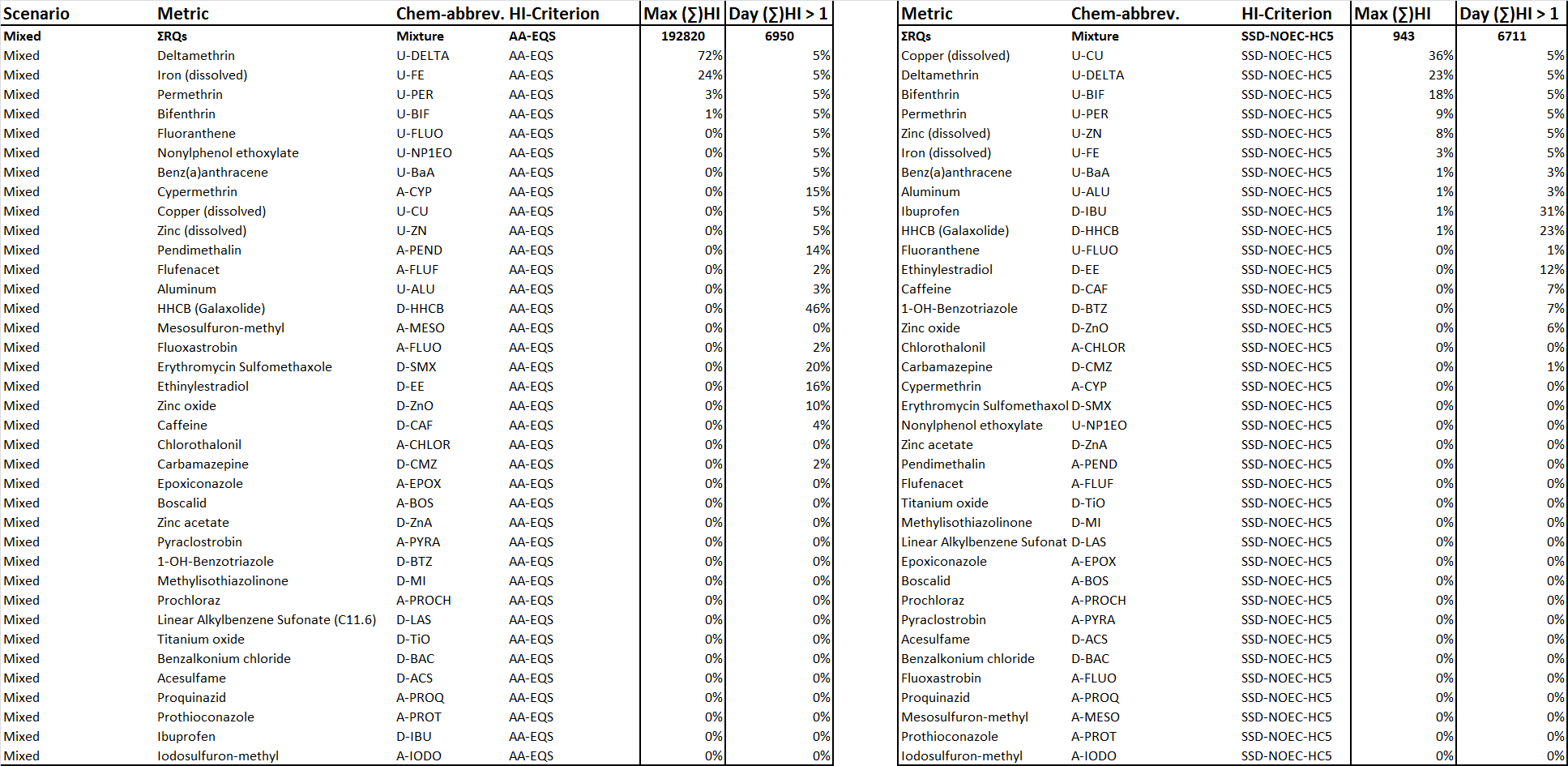


S.I. Table 11. Prioritization of the relative role of chemicals in mixtures, explored via different prioritization questions, e.g., regarding the use of regulatory criteria or ecotoxicological endpoints (left and right tables) and regarding relative contributions of chemicals during peak exposures (left column within tables) or relative frequency of a compound on multiple days when HI>1 was observed.

# Acknowledgments

European surface water concentration data were downloaded from the NORMAN EMPODAT database (<http://www.norman-network.net/empodat/>).

# References

[1] Holmes C, Hamer M, Brown C, Jones R, Weltje L, Maltby L, Posthuma L, Silberhorn E, Teeter S, Warne MSJ. Submitted for the ET&C-Pellston mixture workshop series. Risk assessment of mixtures from agricultural chemicals – Simplifying prospective and retrospective approaches.

[2] Diamond J, et al. Submitted for the ET&C-Pellston mixture workshop series. Use of prospective and retrospective risk assessment methods that simplify chemical mixtures associated with treated domestic wastewater discharges

[3] De Zwart D, Adams W, Galay Burgos M, Hollender J, Junghans M, Merrington G, Muir D, Parkerton T, De Schamphelaere KAC, Whale G, Williams R. Submitted for the ET&C-Pellston mixture workshop series. Aquatic exposures of chemical mixtures in urban environments: approaches to impact assessment

[4] EC. 2003. Proposal for a regulation of the European Parliament and of the Council concerning the Registration, Evaluation, Authorisation and Restriction of Chemicals (Reach), establishing a European Chemicals Agency and amending Directive 1999/45/EC and Regulation (EC) on Persistent Organic Pollutants. European Commission, Brussels, Belgium.

[5] EC. 2000. Directive 2000/60/EC of the European parliament and of the council of 23 October 2000 establishing a framework for Community action in the field of water policy. *Official Journal of the European Communities L* 327:1-72.

[6] Van Straalen NM, Denneman CAJ. 1989. Ecotoxicological evaluation of soil quality criteria. *Ecotoxicology and Environmental Safety* 18:241-251.

[7] Posthuma L, De Zwart D. 2014. Species Sensitivity Distributions. *Encyclopedia of Toxicology, 3rd edition*. Vol 4. Elsevier Inc., Academic Press, pp 363–368.

[8] Price PS, Han X. 2011. Maximum cumulative ratio (MCR) as a tool for assessing the value of performing a cumulative risk assessment. *International Journal of Environmental Research and Public Health* 8:2212-2225.

[9] Malaj E, von der Ohe PC, Grote M, Kühne R, Mondy CP, Usseglio-Polatera P, Brack W, Schäfer RB. 2014. Organic chemicals jeopardize the health of freshwater ecosystems on the continental scale. *Proceedings of the National Academy of Sciences* 111:9549–9554.

[10] Posthuma L, De Zwart D, Osté L, Van der Oost R, Postma J. 2016. Water System Analysis with the Ecological Key Factor "Toxicity". Part 1: The approach, its underpinning and its utility. STOWA, Amersfoort, the Netherlands.

[11] De Zwart D. 2002. Observed regularities in SSDs for aquatic species. In Posthuma L, Suter GW, II, Traas TP, eds, *Species sensitivity distributions in ecotoxicology*. Lewis Publishers, Boca Raton, FL, USA, pp 133-154.

[12] Drescher K, Bödeker W. 1995. Assessment of the combined effects of substances - the relationship between concentration addition and independent action. *Biometrics* 51:716-730.

[13] Posthuma L, De Zwart D. 2012. Predicted mixture toxic pressure relates to observed fraction of benthic macrofauna species impacted by contaminant mixtures. *Environmental Toxicology and Chemistry* 31:2175–2188.

[14] Reilly TJ, Smalling KL, Orlando JL, Kuivila KM. 2012. Occurrence of boscalid and other selected fungicides in surface water and groundwater in three targeted use areas in the United States. *Chemosphere* 89:228-234.

[15] Pomati F, Jokela J, Castiglioni S, Thomas MK, Nizzetto L. 2017. Water-borne pharmaceuticals reduce phenotypic diversity and response capacity of natural phytoplankton communities. *PLOS ONE* 12:e0174207.
